# Multi‐Omics Analysis by Machine Learning Identified Lysophosphatidic Acid as a Biomarker and Therapeutic Target for Porcine Reproductive and Respiratory Syndrome

**DOI:** 10.1002/advs.202402025

**Published:** 2024-07-08

**Authors:** Hao Zhang, Fangyu Hu, Ouyang Peng, Yihui Huang, Guangli Hu, Usama Ashraf, Meifeng Cen, Xiaojuan Wang, Qiuping Xu, Chuangchao Zou, Yu Wu, Bibo Zhu, Wentao Li, Qunhui Li, Chujun Li, Chunyi Xue, Yongchang Cao

**Affiliations:** ^1^ Sate Key Laboratory of Biocontrol School of Life Sciences Sun Yat‐sen University Guangzhou 510006 China; ^2^ Department of Medicine Division of Infectious Diseases Stanford University Stanford CA 94305 USA; ^3^ Bioinformatics and Omics Center Sun Yat‐Sen Memorial Hospital Sun Yat‐Sen University Guangzhou 510120 China; ^4^ Guangdong Provincial Key Laboratory of Malignant Tumor Epigenetics and Gene Regulation Sun Yat‐sen Memorial Hospital Sun Yat‐sen University Guangzhou 510120 China; ^5^ Guangdong Enterprise Key Laboratory for Animal Health and Environmental Control Wen's Foodstuff Group Co. Ltd Yunfu 527439 China; ^6^ National Key Laboratory of Agricultural Microbiology Huazhong Agricultural University Wuhan 430070 China; ^7^ Laboratory of Animal Virology College of Veterinary Medicine Huazhong Agricultural University Wuhan 430070 China; ^8^ The Cooperative Innovation Center for Sustainable Pig Production Huazhong Agricultural University Wuhan 430070 China

**Keywords:** antiviral approach, diagnostic biomarker, immune suppression, lysophosphatidic acid (LPA), machine learning, multi‐omics, porcine reproductive and respiratory syndrome virus

## Abstract

As a significant infectious disease in livestock, porcine reproductive and respiratory syndrome (PRRS) imposes substantial economic losses on the swine industry. Identification of diagnostic markers and therapeutic targets has been a focal challenge in PPRS prevention and control. By integrating metabolomic and lipidomic serum analyses of clinical pig cohorts through a machine learning approach with in vivo and in vitro infection models, lysophosphatidic acid (LPA) is discovered as a serum metabolic biomarker for PRRS virus (PRRSV) clinical diagnosis. PRRSV promoted LPA synthesis by upregulating the autotaxin expression, which causes innate immunosuppression by dampening the retinoic acid‐inducible gene I (RIG‐I) and type I interferon responses, leading to enhanced virus replication. Targeting LPA demonstrated protection against virus infection and associated disease outcomes in infected pigs, indicating that LPA is a novel antiviral target against PRRSV. This study lays a foundation for clinical prevention and control of PRRSV infections.

## Graphical abstract

This study analyzes metabolomic and lipidomic profiles of PRRSV‐infected pigs using a machine learning approach, identifying LPA as a serum diagnostic biomarker. PRRSV augments LPA synthesis by upregulating ATX expression, leading to RIG‐I and IFN‐β suppression, and increases virus replication. Targeting LPA alleviates clinical symptoms, reduces viral loads, and mitigates viral shedding.

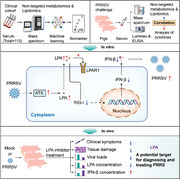

## 1 Introduction

Porcine reproductive and respiratory syndrome (PRRS), stemming from infection with porcine reproductive and respiratory syndrome virus (PRRSV), manifests as a highly contagious swine infectious ailment known for its capacity to induce host immune suppression. This malady exerts a substantial economic burden, resulting in annual production losses approaching one billion USD within the swine husbandry sector.^[^ ^1^ ^]^ Clinical diagnosis of PRRS in pig populations is initially based on the manifestation of symptoms. However, variations in the rearing environments across different swine farms and the diverse health conditions of pig herds contribute to varying clinical presentations following PRRSV infection. Moreover, infections by other pathogens, such as pseudorabies virus (PRV), *Actinobacillus pleuropneumoniae*, and others, can induce similar clinical symptoms, which hinders accurate clinical diagnosis.^[^ ^2^, ^3^, ^4^ ^]^

Currently, conventional laboratory diagnostic methods remain predominant in the clinical diagnosis of PRRSV. Egli et al. established a fluorescence reverse transcription‐polymerase chain reaction (RT‐PCR) method capable of rapid discrimination between PRRSV type 1 and type 2 strains with high specificity and sensitivity.^[^ ^5^ ^]^ Sorensen et al. introduced a blocking enzyme linked immunosorbent assa (ELISA) for high‐throughput PRRSV antibody detection in serum samples.^[^ ^6^, ^7^ ^]^ Additionally, oral fluid can be used for antibody detection with high sensitivity and specificity compared to serum.^[^ ^8^, ^9^, ^10^, ^11^ ^]^ It has been widely employed for population‐level PRRSV antibody monitoring in sow herds and growing pig populations.^[^ ^9^, ^12^, ^13^ ^]^ However, to date, there has been a lack of development and application of diagnostic biomarkers specific to PRRSV.

Presently, the clinical management of PRRS primarily relies upon palliative interventions aimed at symptomatic relief, given the absence of effective pharmacological treatments. Moreover, vaccine‐based immunization strategies face formidable challenges attributable to the marked variability exhibited by PRRSV.^[^ ^14^ ^]^ Therefore, there exists an urgent need for the identification of precise therapeutic targets endowed with the capacity to combat PRRSV infection in a highly specific manner.^[^ ^15^, ^16^ ^]^

During viral infections, viruses can induce metabolic alterations in infected host cells. Manipulating these changes holds promise for altering the outcomes of viral infections, thereby offering potential targets for therapies aimed at inhibiting viral infection or replication. For instance, influenza virus, dengue virus, and severe acute respiratory syndrome coronavirus 2 (SARS‐CoV‐2) have been observed to induce altered glucose and lipid metabolism in infected cells.^[^ ^17^, ^18^, ^19^, ^20^ ^]^ In the case of hepatitis B virus, the generation of lactate, dependent on lactate dehydrogenase A, suppresses RIG‐I‐like receptors (RLR) signaling, which in turn leads to immune evasion.^[^ ^21^ ^]^ Targeting these metabolic pathways has been found as an effective strategy to restrain viral replication. Clinical investigations of coronavirus disease 2019 (COVID‐19) have unveiled close associations between circulating serum metabolites, inflammatory cytokines, and the immune competence of patients, potentially identifying them as promising therapeutic targets.^[^ ^22^ ^]^

During PRRSV infection, it has been shown that PRRSV infection actively promotes glycolytic metabolism that contributes to the maintenance of the tricarboxylic acid (TCA) cycle.^[^ ^23^ ^]^ Analysis of serum samples obtained from PRRSV‐infected pregnant sows suggests that certain molecules, namely alpha‐AAA, kynurenine, and lysoPCs, hold promise as crucial biomarkers associated with PRRSV infection.^[^ ^24^ ^]^ These findings strongly advocate for the potential of metabolic modulation as a pivotal avenue for therapeutic interventions against PRRSV.

In this study, by integrating clinical cohorts, multi‐omics analyses, and molecular mechanistic approaches, we identified and subsequently validated lysophosphatidic acid (LPA) as a prospective target for both diagnostic and therapeutic purposes in the context of PRRSV. Importantly, we provided compelling evidence confirming the efficacy of LPA‐targeting as a novel treatment of PRRSV. This study may provide new opportunities for the diagnosis and eradication of PRRSV in swine population.

## 2 Results

### 2.1 Utilizing Machine Learning for the Identification of Metabolic and Lipid Serum Biomarkers in a Clinical Cohort of PRRSV‐Infected Pigs

**Figure 1:**
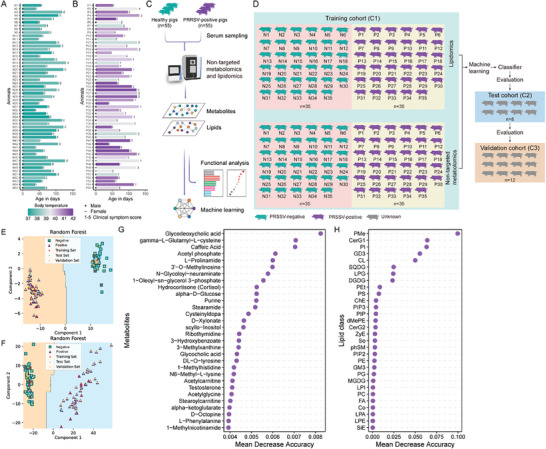
Machine learning‐based discrimination of PRRSV‐infected and uninfected pigs using serum metabolomic and lipidomic features of a clinical cohort of pigs. A,B) Clinical profile of each animal recruited into the study. The Y‐axis represents individual animals, labeled N1–N55 for PRRSV‐negative (A) and P1–P55 for PRRSV‐positive (B) animals. The X‐axis denotes the age of the animals in days. The color of each bar indicates the body temperature of the pigs. Gender is represented by a “+” (male) or “–” (female) symbol, while clinical symptom scores are denoted by numerical values to the right of each bar. C) Overview of metabolic and lipidomic workflow. Clinical serum samples from pigs were collected and assessed for PRRSV positivity using RT‐qPCR, followed by classification into PRRSV‐positive or negative groups (*n* = 55 per group). Subsequent analysis included untargeted metabolomic and lipidomic analyses of each serum sample with the implication of data for functional study and machine learning. D) Protocol for developing a machine learning‐based classifier for PRRSV detection. A training cohort (C1) was established for initial sample acquisition and untargeted metabolomic and lipidomic profiling. The classifier's performance was subsequently assessed in an independent test cohort (C2), followed by further validation in a validation cohort (C3). E,F) A random forest analysis in machine learning to globally analyze untargeted metabolomic (E) and lipidomic (F) data. G,H) Top 30 candidate biomarker metabolites (G) or lipids (H) identified through machine learning.

We employed a swine cohort to investigate the metabolic biomarkers in the serum samples of pigs infected with PRRSV. To do so, a cohort of a total of 110 animals, including PRRSV‐positive (*n* = 55) and negative matched controls (*n* = 55), were selected based on a quantitative reverse transcription polymerase chain reaction (RT‐qPCR) detection method, presence of PRRSV‐specific clinical signs, and absence of other relevant and common pig diseases. All animals were kept under monitoring for a change in body temperature (**Figure** **1**A,B) and the appearance of PRRSV‐specific clinical symptoms before the collection of serum samples (Figure 1B). Subsequently, serum samples were subjected to untargeted metabolomic and lipidomic analyses separately by using a mass spectrometry approach (Figure 1C). A protocol for developing a random forest machine learning‐based classifier for PRRSV detection is shown in Figure 1D. A training cohort (C1; *n* = 35) was established for initial sample acquisition and untargeted metabolomic and lipidomic profiling. The classifier's performance was subsequently assessed in an independent test cohort (C2; *n* = 8), followed by further validation in a validation cohort (C3; *n* = 12). A random forest algorithm of machine learning and principal component analysis (PCA) revealed a notable distinction among the tested groups (Figure 1E,F; Figure S1A,C, Supporting Information). The efficiency of machine learning in analyzing untargeted metabolomic and lipidomic data was confirmed by generating the receiver operating characteristic (ROC) curves, the model reached an area under curve (AUC) of 1.00 both in the untargeted metabolic and lipidomic training set (Figure S1B,D, Supporting Information). The significance of the predicted results was exceptionally high, with p‐values of 2.0e‐9 and 3.2e‐9 for the untargeted metabolomic and lipidomic prediction models, respectively, as determined by the Chi‐square test. The utilization of this machine learning approach led us to identify biomarkers related to metabolites (Figure 1G) and lipids (Figure 1H) that showed a significant alteration in the serum of PRRSV‐infected pigs than uninfected controls. Moreover, the pathway enrichment analyses of differentially expressed serum biomarkers revealed their association with cellular pathways related, but not limited, to glucose and lipid metabolisms (Figure S1E,F, Supporting Information). Overall, these analyses identify serum metabolic and lipid biomarkers that undergo differential alteration in a clinical cohort of PRRSV‐infected pigs.

### 2.2 PRRSV Infection Induces a Host Reprogramming of Glucose and Lipid Metabolism

**Figure 2:**
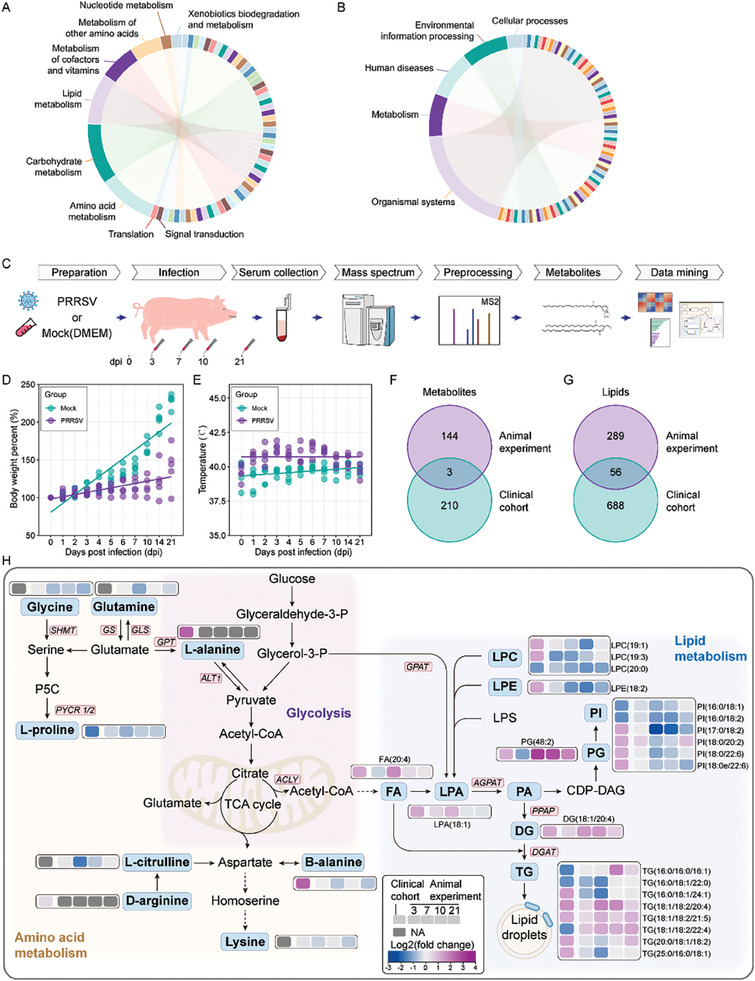
Metabolic and lipidomic analysis in animal virus‐challenged experiments reflecting similarities to data in a clinical cohort. A,B) Classification of KEGG pathways related to metabolites (A) and lipids (B) detected in untargeted metabolomic (A) and lipidomic (B) data from the clinical cohort. C) Overview of study design for PRRSV challenge in a piglet model (*n* = 5 for each group). D,E) Changes in piglet body weight (D) and temperature (F) post‐infection. In D, the initial weight at 0 dpi was set as 100%, and subsequent changes were calculated relative to this value. F,G) Venn diagrams illustrate an overlap of differentially expressed metabolites (F) and lipids (G) between virus‐challenged animal experiment and clinical cohort, calculated and generated using online tool Jvenn.^[^ ^48^ ^]^ H) Composite diagram highlighting shared differentially expressed metabolites and lipids in both animal experiment and clinical cohort. Expression values are depicted in terms of Log2 fold‐change. The integrated figure was plotted using Adobe Illustrator.

To further elucidate the differential serum metabolites and lipids in clinical cohort samples, we then classified enriched pathways related to untargeted metabolites and lipids, which were found to converge on lipid metabolism, glucose metabolism, carbon metabolism, and their corresponding biological functions (**Figure** **2**A,B; Table S1, Supporting Information). To corroborate the clinical cohort findings, we employed a PRRSV challenge animal model, categorized into infected and uninfected groups. Serum samples were collected at 3, 7, 10, and 21 days post‐infection (dpi) for untargeted metabolomic and lipidomic analyses using mass spectrometry (Figure 2C). Concurrently, ongoing monitoring of body temperature, body weight, clinical symptoms, and nasal viral shedding in the infected and uninfected groups revealed significant disparities. The infected animals exhibited pronounced clinical symptoms, including high fever and weight loss (Figure 2D,E; Figure S2A, Supporting Information) together with high viral loads in nasal swabs (Figure S2B, Supporting Information).

Upon analyzing the metabolomic data, PCA result demonstrated robust inter‐group distinctiveness and inner‐group reproducibility (Figure S2C,D, Supporting Information). In addition, a statistical enumeration of differentially expressed metabolites and lipids was conducted (Figure S2E–J, Supporting Information) and compared with the findings from the clinical cohort which showed a small overlap for both metabolites and lipids (Figure 2F,G). Under controlled PRSSV challenge settings, infected animals showed obvious differences in the levels of metabolites and lipids when compared to the animals belonging to the uninfected group (Figure S2G,H, Supporting Information). Notably, the smallest number of differentially expressed metabolites was observed at 3 dpi, whose numbers increased markedly over time (Figure S2E,F, Supporting Information). The number of differential metabolites showing an intersection at different infection time points is negligible (Figure S2I, Supporting Information); however, that intersection proportion increased significantly for the differentially expressed lipids (Figure S2J, Supporting Information). Furthermore, a temporal cluster enrichment analysis was performed to examine the variation in the expression of metabolites and lipids over time (Figure S3, Supporting Information). Both types of molecules were clustered into eight distinct clusters by considering their expression tendencies along with the time. The metabolites (Figure S3A, Supporting Information) and lipids (Figure S3B, Supporting Information) that clustered together are more likely to be classified into the same functional set of molecules. The roles of differentially expressed metabolites and lipids were further elucidated through functional enrichment analysis (Figure S2K,L, Supporting Information), highlighting their respective significance. Presentation of the common differentially expressed metabolites and lipids shared between the clinical cohort and the challenge model, including pathway annotations and relative expression levels, revealed a homology of these metabolites in lipid droplet metabolism, glucose metabolism, and amino acid metabolism (Figure 2H), aligning with the previously observed enrichment results in the clinical cohort.

### 2.3 LPA and IFN‐β Exhibit a Strong Inverse Correlation in Their Regulation

**Figure 3:**
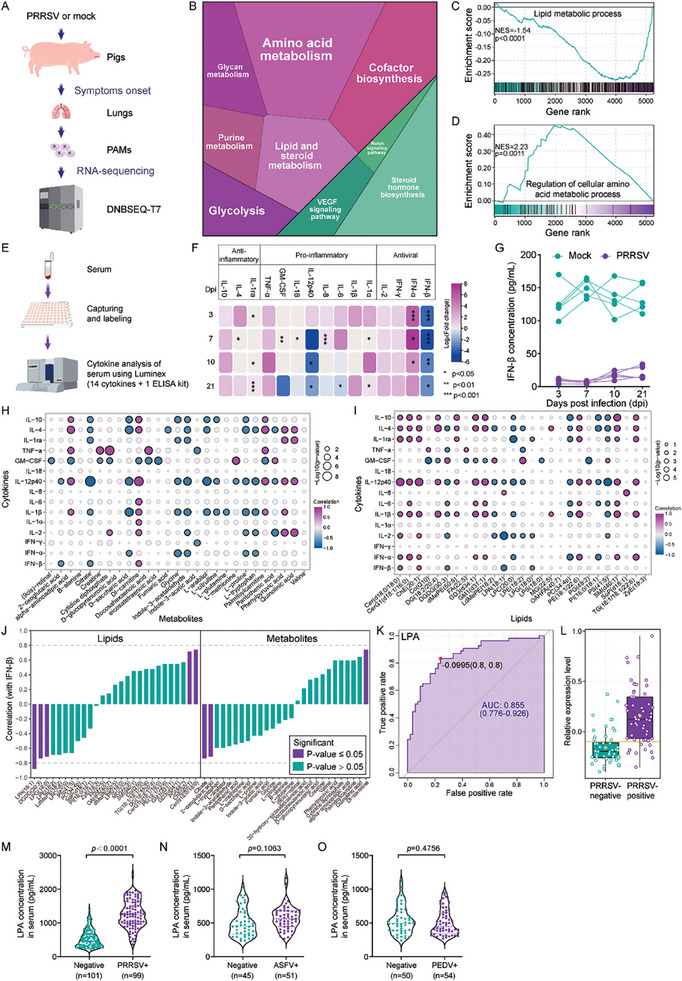
Correlation analysis reveals LPA as a potential biomarker and immune modulator during PRRSV infection of piglets. A) Schematic of the study design for RNA‐sequencing (*n* = 3 per group). B) KEGG pathway analysis of differentially expressed genes (DEGs) in metabolic processes. Diagram was generated by utilizing Proteomaps.^[^ ^49^ ^]^ C,D) Gene set enrichment analysis (GSEA) of RNA‐sequencing data highlighting the significance of lipid (C) and amino acid (D) metabolism during PRRSV infection. The analysis was performed with GSEA database.^[^ ^50^ ^]^ E) Schematic of cytokine detection in serum samples obtained from animal virus‐challenged experiments at 3, 7, 10, and 21 days post‐infection (dpi). F) Comparative fold change of cytokines showing differential expression in infected animals compared to mock‐infected animals. G) Measurement of IFN‐β protein levels in the serum samples of PRRSV‐infected and mock‐infected individual animals. H,I) Heatmaps showing correlations between cytokines and metabolites (H) or lipids (I). J) Targeted correlation analysis between IFN‐β and both metabolites and lipids. K) ROC curve analysis of LPA as identified by machine learning. L) Relative expression levels of LPA in a in lipidomic data of the clinical pig cohort (*n* = 110) related to Figure 1. M–O) LPA concentrations in the serum of PRRSV‐infected (M) pigs compared to ASFV‐ (N) or PEDV‐infected (O) pigs recruited in expanded clinical cohorts. Significant comparisons between two groups are made using Mann‐Whitney test.

Given that the primary target cells of PRRSV are the porcine alveolar macrophages (PAMs), we sought whether there is a concordance in the metabolic reprogramming outcomes between the target cells and the serum. To address this, we employed a PRRSV infection animal model, followed by utilizing PAMs isolated from porcine lungs for RNA sequencing (**Figure** **3**A). PCA result of sequencing data confirmed consistent reproducibility within groups and obvious distinctions between infected and non‐infected groups (Figure S4A, Supporting Information). The differentially expressed genes (DEGs) were subsequently identified and illustrated in a volcano plot wherein the majority of DEGs showed an upregulated expression pattern (Figure S4B,C, Supporting Information). Pathway enrichment analyses revealed metabolism as one of the highly enriched pathways related to DEGs (Figure S4D, Table S3, Supporting Information). A further characterization of the highly enriched metabolism pathway indicated its relevance primarily to lipid, amino acid, glycan, and glucose metabolism (Figure 3B). Consistently, the gene set enrichment analysis (GSEA) also suggested the DEGs enrichment in lipid and amino acid metabolism (Figure 3C,D), mirroring the serum enrichment results. We next assessed the cytokine profile in the serum of the PRRSV‐challenged animal model at multiple infection time points (Figure 3E) and evaluated the correlation between cytokines and metabolites or lipids. The cytokines belonging to anti‐inflammatory, pro‐inflammatory, and anti‐viral categories indicated notable changes in their expression levels upon infection (Figure 3F). Among them, interferon beta (IFN‐β) exhibited the most significant downregulation at all infection time points (Figure 3F), which was further confirmed to be consistently lowered in the serum of infected animals individually compared to non‐infected animals (Figure 3G). Correlation analysis of cytokines with metabolites (Figure 3H) or lipids (Figure 3I) indicated the strongest negative correlation of IFN‐β with lysophosphatidic acid (LPA) (Figure 3J). Subsequently, the ROC curve analysis of a clinical pig cohort data by machine learning unveiled LPA as a potential clinical serum biomarker (Figure 3K), exhibiting a significant upregulation in the infected group (Figure 3L). LPA, as well as phosphatidylinositol (PI), were also implicated as the highest number of pathways related to lipid metabolism (Figure S4E, Supporting Information), which highlights a multifaceted role of LPA in the PRRSV pathogenesis. The elevated levels of serum LPA were also assessed by recruiting an additional cohort of PRRSV‐infected subjects (*n =* 200) which revealed a significant upregulation in LPA content in the serum of PRRSV‐positive subjects compared to the negative animals (Figure 3M). However, no significant differences were observed in the serum of clinical cohorts of African swine fever virus (ASFV)‐ and porcine epidemic diarrhea virus (PEDV)‐ positive subjects (Figure 3N,O).

Taken together, these data indicate that PRRSV infection increases the serum level of LPA which negatively correlates with the cytokines production in vivo.

### 2.4 LPA Promotes PRRSV Replication by Suppressing IFN‐β Production In Vitro

**Figure 4:**
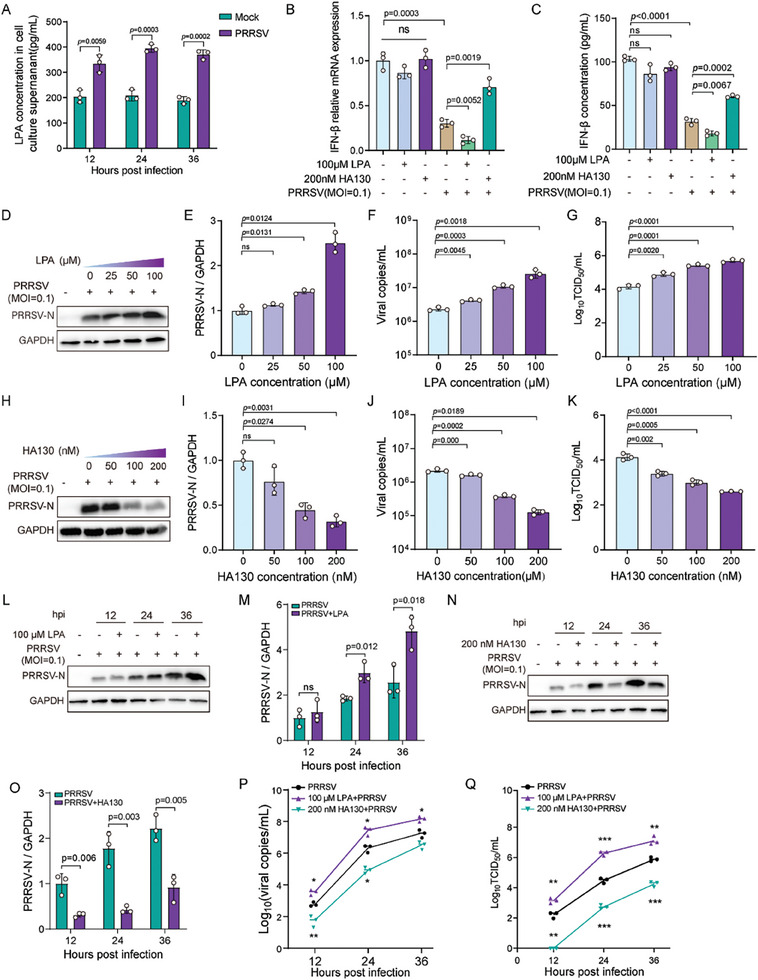
LPA promotes PRRSV replication by suppressing the host IFN response. A) Confirmation of LPA upregulation in PRRSV‐infected iPAMs cell culture supernatants. Cells were infected with PRRSV at an MOI of 0.1, followed by LPA detection by ELISA in the supernatants at indicated time‐points post‐infection. B,C) Alteration of IFN‐β mRNA (B) and protein (C) expressions in PRRSV‐infected iPAM cells upon supplementation or inhibition of LPA. Cells were treated with LPA supplement or LPA inhibitor HA130 at indicated concentration for a period of 1 h, following infection with PRRSV at indicated MOI. At 24 h post‐infection, IFN‐β mRNA and protein expression were detected by RT‐qPCR and ELISA, respectively. D–G) Exogenous LPA increases PRRSV proliferation in iPAM cells evaluated at the level of viral protein (D and E), viral copy number (F), and viral titers (G) in a dose‐dependent manner. H–K) Inhibition of LPA reduces PRRSV proliferation in iPAM cells evaluated at the level of viral protein (H and I), viral copy number (J), and viral titers (K) in a dose‐dependent manner. L–Q) Exogenous LPA enhances PRRSV proliferation in iPAM cells evaluated at the level of viral protein (L and M), viral copies (P), and viral titers (Q) in a time‐dependent manner. LPA inhibition reduces PRRSV replication in iPAM cells evaluated at the level of viral protein (N and O), viral copy number (P), and viral titers (Q) in a time‐dependent manner. In D–Q, cells were treated with indicated concentration of exogenous LPA or HA130 for a period of 1 h, followed by PRRSV infection at indicated MOI and time. The biological experiments were conducted in triplicate. Data are represented as mean ± SEM. Significant comparisons between two groups are made using Mann‐Whitney test, significant comparisons among three or more groups are made by one‐way ANOVA, with significance levels indicated as ns (not significant), * (*p* < 0.05), ** (*p* < 0.01), and *** (*p* < 0.001).

**Figure 5:**
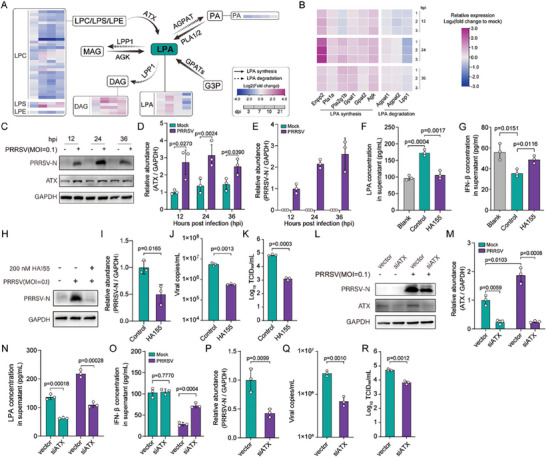
PRRSV promotes LPA synthesis via upregulation of ENPP2 (ATX). A) Heatmap showing the expression levels of lipids, associated with LPA synthesis and degradation, in animal virus‐challenged experiment data. B) RT‐qPCR analysis of mRNA expression of genes related to LPA synthesis and degradation in PRRSV‐infected iPAM cells. C–E) Confirmation of ATX upregulation at protein level following PRRSV infection in iPAM cells. F–K) HA155‐mediated inhibition of ATX decreases LPA synthesis, alleviates IFN‐β suppression and reduces the PRRSV proliferation in iPAM cells. L–R) siRNA‐mediated inhibition of ATX decreases LPA synthesis, alleviates IFN‐β suppression and reduces the PRRSV proliferation in iPAM cells. In F–R, cultured iPAM cells were subjected to treatment with HA155 or siATX for either 1 hour or 24 hours, along with their respective controls, followed by 0.1 MOI infection of PRRSV for a period of 24 h. LPA and IFN‐β levels were determined by ELISA. The viral replication at the level of protein, genome, and titers was determined by Western blot, RT‐qPCR, and plaque assay, respectively. The biological experiments were conducted in triplicate. Data are represented as mean ± SEM. Significant comparisons between two groups are made using Mann‐Whitney test, significant comparisons among three or more groups are made by one‐way ANOVA.

Considering that LPA and IFN‐β show significantly altered expressions upon PRRSV infection in vivo and their expressions are inversely related to each other, we further investigated their impact on PRRSV replication using an in vitro culture system. To this end, immortalized porcine alveolar macrophage (iPAM) cells were infected with PRRSV at a multiplicity of infection (MOI) of 0.1, and the production of LPA was determined using ELISA. PRRSV infection of cultured cells led to markedly increased levels of LPA in a time‐dependent manner when compared to non‐infected cells (**Figure** **4**A). We next examine the effect of LPA supplementation and its inhibition on IFN‐β production and virus replication. It was found that the treatment of infected cells with non‐cytotoxic concentrations of LPA supplement (Figure 4B,C; FigureS5A, Supporting Information) resulted in a significantly reduced production of IFN‐β at both RNA and protein levels and enhanced virus replication in an LPA supplement dose‐dependent manner as assessed by quantifying viral protein (Figure 4D,E), genome copies (Figure 4F), and titers (Figure 4G). However, the non‐cytotoxic concentrations of LPA inhibitor HA130, inhibiting LPA production in the culture supernatants effectively (Figure S5B,C, Supporting Information), caused an opposite effect on both IFN‐β levels (Figure 4B,C) and virus replication (Figure 4H–K) in treated cells. Similar effects on IFN‐β levels and virus replication were observed when a single non‐cytotoxic concentration of LPA supplement and inhibitor HA130 were tested in an infection time‐dependent manner (Figure 4L–Q; Figure S5D–F, Supporting Information). Furthermore, the effect of LPA supplementation and inhibition on pro‐inflammatory, anti‐inflammatory, and antiviral cytokines was evaluated. None of the cytokines tested, except IL‐1β, at both RNA and protein levels, demonstrated a strong correlation with the LPA under infection conditions (Figure S6, Supporting Information). Altogether, these findings indicate that LPA enhances PRRSV replication by dampening the host IFN‐β response in infected cells.

### 2.5 LPA Synthesis is Promoted by the Upregulation of ENPP2 (ATX) Following PRRSV Infection

To elucidate the underlying mechanisms of PRRSV‐mediated upregulation of LPA, we analyzed metabolomic data from the PRRSV‐challenged in vivo model pertaining to metabolites associated with LPA decomposition and synthesis (**Figure** **5**A). Concurrently, we conducted the RT‐qPCR analysis of genes related to LPA decomposition and synthesis. In addition to several genes showing a significantly perturbed expression, the levels of ectonucleotide pyrophosphatase 2 (ENPP2, also called autotaxin or ATX), one of the genes involved in LPA synthesis, showed a significant increase at all three infection time points following PRRSV infection (Figure 5B). Protein‐level quantification corroborated these findings, demonstrating consistency with the mRNA expression levels (Figure 5C–E). Furthermore, the treatment of cells with an ATX inhibitor (HA155) resulted in a significant reduction in LPA levels in the cell supernatants, concomitant with the restoration of IFN‐β levels and pronounced inhibition of PRRSV replication (Figure 5F–K). Similar outcomes were observed when ATX expression levels were knocked down using the ATX‐specific siRNAs (Figure 5L–R). In contrast, the overexpression of ATX exhibited a modest promotion of LPA levels in the cell supernatant, albeit insignificantly, and demonstrated no significant impact on IFN‐β level and viral replication (Figure S7, Supporting Information). Thus, these data demonstrate that PRRSV augments LPA synthesis via upregulating the ATX production in infected cells.

### 2.6 LPA Inhibits IFN‐β Response by Suppressing RIG‐I Expression Following PRRSV Infection

**Figure 6:**
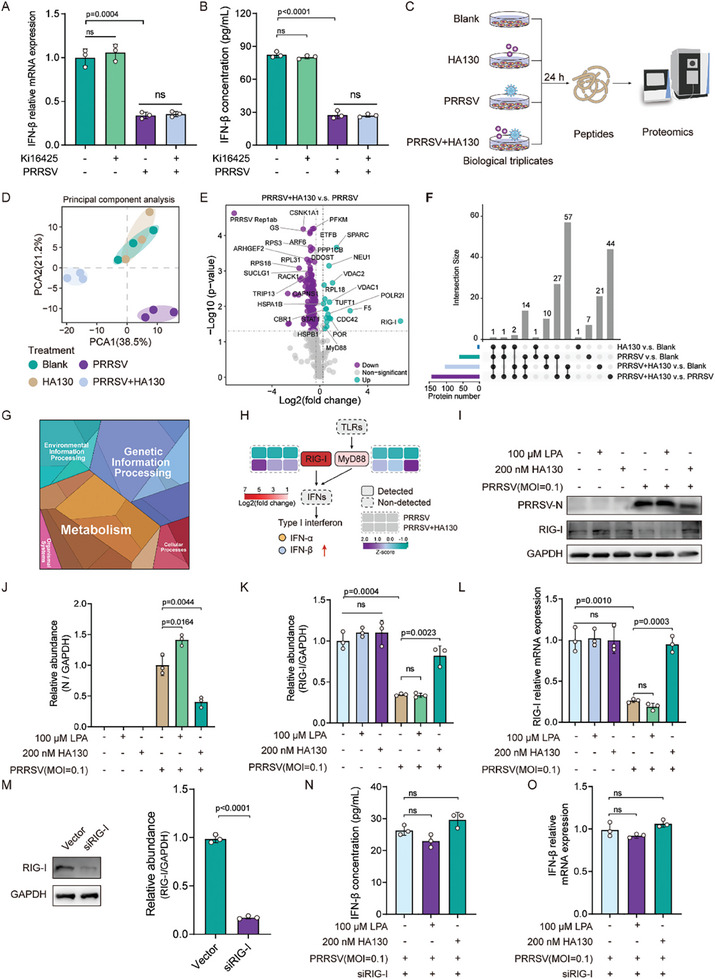
LPA suppresses IFN‐β response by dampening the RIG‐I expression during PRRSV infection. A,B) LPAR1 inhibition in iPAM cells does not affect IFN‐β suppression during PRRSV infection. C–H) Proteomic analysis of IFN‐β inhibition by PRRSV in iPAM cells: iPAMs treatment and collection for proteomic sequencing (C); PCA analysis showing strong internal group replication (D); identification of differentially expressed proteins (DEPs) (E); heatmap of DEPs in PRRSV+HA130 group relative to PRRSV group (F); KEGG functional enrichment analysis of DEPs (G); expression levels of proteins associated with IFN generation (H). I–L) LPA‐mediated regulation of RIG‐I expression. Cultured iPAM cells were treated with indicated concentrations of LPA or HA130, followed by PRRSV infection at an MOI of 0.1 for 24 h. The protein and mRNA levels of viral N‐protein and RIG‐I were determined by Western blot and RT‐qPCR. M–O) Effect of RIG‐I inhibition on IFN‐β production. Cultured iPAM cells, treated with siRIG‐I, LPA, HA130, or their controls for 1 h, were subjected to PRRSV infection at MOI of 0.1 for 24 h. The effect of siRNAs on RIG‐I protein expression was assessed by Western blot. The proteins and mRNA levels of IFN‐β were quantified by ELISA and RT‐qPCR, respectively. The biological experiments were conducted in triplicate. Data are represented as mean ± SEM. Significant comparisons between two groups are made using Mann‐Whitney test, significant comparisons among three or more groups are made by one‐way ANOVA, with significance levels indicated as ns (not significant).

It is reported that LPA regulates interferon regulatory factor 3 (IRF3) via the LPA1 receptor, thereby inhibiting the levels of IFN‐β.^[^ ^25^ ^]^ To confirm whether this phenomenon also occurs in the case of PRRSV infection, iPAM cells, treated with or without an LPA1 inhibitor (Ki16425), were subjected to PRRSV infection followed by quantification of IFN‐β levels. Inhibition of LPA1 caused no effect on IFN‐β production at both mRNA and protein levels (**Figure** **6**A,B) without altering cell viability (Figure S8A, Supporting Information) and virus replication (Figure S8B,C, Supporting Information). To elucidate the mechanism by which LPA negatively regulates IFN‐β response under PRRSV infection, the LPA inhibitor HA130 treated or untreated iPAM cells were infected with PRRSV, and protein samples were harvested at 24 hpi for proteomic analysis (Figure 6C). PCA analysis of proteomics data showed notable distinctions between the groups and similarities within the groups (Figure 6D). Differentially expressed protein (DEPs) analysis, shown as volcano plots, revealed the perturbation of several cellular proteins dependent on tested experimental conditions (Figure 6E; Figure S8D–F, Supporting Information), wherein the HA130 treated and infected cells predominantly showed a differential downregulated DEPs (Figure 6E,F). The pathway enrichment analysis of DEPs in HA130‐treated and infected cells indicated their enrichment primarily in metabolism pathways (Figure 6G). Further analysis of the IFN pathway revealed indicated the marked downregulation of retenoic inducible gene‐I (RIG‐I) and myeloid differentiation primary response protein 88 (MyD88) levels upon PRRSV infection, whose expression rescued in HA130‐treated cells (Figure 6H), indicating that the expression of RIG‐I and MyD88 is regulated by LPA. This phenotype was confirmed in vitro where the exogenous treatment of LPA suppressed RIG‐I expression at both RNA and protein levels in cultured cells, whereas LPA inhibition had the opposite effect (Figure 6I–L). Similar results were observed for the MyD88, though the regulatory effect of LPA on MyD88 was not as pronounced as on RIG‐I (Figure S8G–I, Supporting Information). Subsequently, silencing RIG‐I expression or modulating LPA at the cellular level resulted in no significant regulatory effect on IFN‐β (Figure 6M–O), suggesting that LPA regulates IFN‐β by suppressing RIG‐I expression. The mechanism by which LPA suppresses RIG‐I at the mRNA and protein levels remains unclear. Interferon regulatory factor 1 (IRF1) is known to activate the RIG‐I promoter, thus regulating RIG‐I transcription.^[^ ^26^ ^]^ To investigate this, we isolated and analyzed proteins from the nucleus and cytoplasm. Our results showed that PRRSV infection prevents IRF1 to translocate from the cytoplasm to the nucleus. LPA exacerbates this effect, whereas inhibition of LPA production restores IRF1's nuclear translocation (Figure S8J–L, Supporting Information). Collectively, these findings suggest that LPA probably suppresses RIG‐I expression by blocking IRF1 nuclear translocation, thereby regulating the IFN‐β response.

### 2.7 Targeting LPA Exhibits Promising Therapeutic Efficacy Against PRRSV Infection

**Figure 7:**
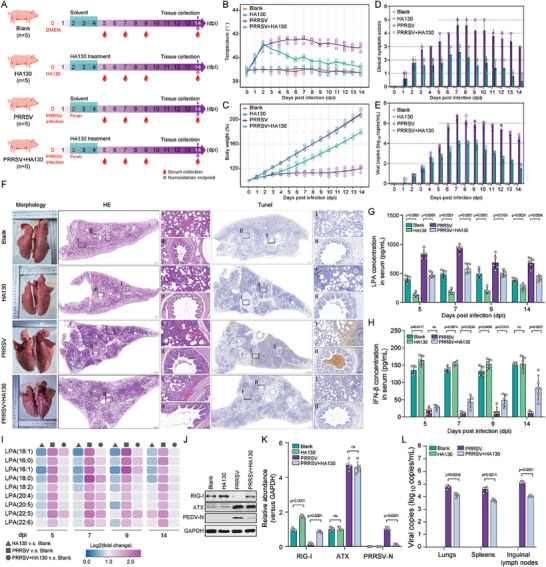
Therapeutic efficacy of HA130 in limiting PRRSV replication and mitigating disease pathogenesis in piglets. A) Overview of experimental design indicating the HA130 treatment and PRRSV infection of piglets. B–E) Effect of HA130 treatment on body temperature (B), body weight (C), clinical symptoms (D), and viral replication (E) in PRRSV‐infected piglets. F) Effect of HA130 treatment on morphology, histology, and apoptosis of lung tissues of infected animals. Histological and apoptotic features were visualized by H&E staining and TUNEL assay, respectively. G–H) ELISA‐based measurement of serum LPA (G) and IFN‐β (H) concentrations at 5, 7, 9, and 14 days post‐infection (dpi). I) Lipidomic sequencing for serum LPA quantification at 5, 7, 9, and 14 dpi. J–K) Western blot analysis of ATX and RIG‐I expression in lung tissues at 14 dpi. L) RT‐qPCR assessment of viral loads in lungs, spleens, and inguinal lymph nodes at 14 dpi. Data are represented as mean ± SEM. Significant comparisons between two groups are made using Mann‐Whitney test, significant comparisons among three or more groups are made by one‐way ANOVA, with significance levels indicated as ns (not significant).

To validate whether targeting LPA can suppress PRRSV replication and ameliorate clinical symptoms, we determined the activity of LPA inhibitor HA130 in an in vivo PRRSV‐challenged model system. All animals (*n* = 5 per group) were classified into the following groups: blank (control group), HA130 (treatment group), PRRSV (infection group), and PRRSV+HA130 (infection‐treatment group) (**Figure** **7**A). Clinical assessments, including clinical scores, body temperature, and weight were conducted. The average body temperature of piglets in PRRSV infection group markedly increased from 38.7 °C at 0 dpi to 41.2 °C at 3 dpi and remained above 40 °C until the observation endpoint. However, treatment with HA130 resulted in a decrease in body temperature to below 40 °C after 7 dpi (Figure 7B). HA130 treatment also significantly promoted body weight gain and mitigated clinical symptoms induced by PRRSV infection (Figure 7C,D). These findings indicated that the treatment with HA130 effectively alleviated the severe fever, weight loss, and clinical disease symptoms in piglets induced by PRRSV infection (Figure 7B–D), which correlated with the limited virus production in the upper respiratory tract as assessed by quantifying virus RNAs in nasal swab samples (Figure 7E). Gross and histopathological analysis of animal tissues, including lungs, spleen, and inguinal lymph nodes, demonstrated a significant amelioration of PRRSV‐mediated pathological changes and apoptosis upon treatment of animals with HA130 (Figure 7F; Figure S9, Supporting Information). Moreover, the treatment of HA130 significantly decreased the levels of LPA and its various subtypes in the serum of treated animals, while markedly restoring the serum IFN‐β levels (Figure 7G–I). The regulatory effect of LPA inhibition on the other cytokines remained modest: interleukin 8 (IL‐8) and IL‐4 showing an upregulation, whereas IFN‐α, IL‐1β, IL‐10, IL‐6, and tumor necrosis factor alpha (TNF‐α) demonstrated a trend of downregulation. (Figure S10, Supporting Information). We finally examined if the observed outcomes are dependent on ATX and RIG‐I expression in lung tissues. Indeed, PRRSV infection significantly enhanced ATX, but reduced RIG‐I, proteins abundance in lung tissues. In contrast, the HA130 treatment of animals led to the rescue of the inhibitory effect of PRRSV on RIG‐I, which ultimately caused a significant suppression of viral loads in the lungs, spleen, and inguinal lymph nodes (Figure 7J–L). Overall, these data suggest that the inhibition of LPA during PRRSV infection exerts an antiviral effect which results in the amelioration of disease outcomes in infected piglets.

## 3 Discussion

**Figure 8:**
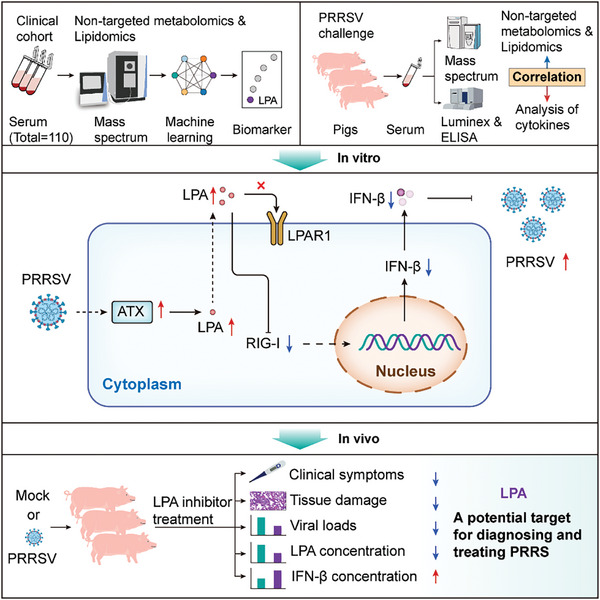
Graphical depiction of the study design and the underlying mechanism by which PPRSV elevates LPA levels to downregulate RIG‐I, thereby inhibiting the production of IFN‐β and promoting the proliferation of PRRSV.

We analyzed metabolomic and lipidomic profiles of clinical cohorts of PRRSV‐infected pigs with a machine learning approach and identified LPA as a serum diagnostic biomarker for PRRSV infection. Mechanistically, PRRSV augmented LPA synthesis by upregulating the ATX expression which led to RIG‐I and IFN‐β suppression and consequently increased virus replication in vitro and in vivo. Targeting LPA proved effective in alleviating clinical symptoms induced by PRRSV, reducing viral loads in various organs, and mitigating viral shedding (**Figure** **8**).

With the emergence of high‐throughput technologies, a novel approach to investigate viral diseases and facilitate diagnosis involves multiomic assessment of clinical cohorts for virus‐specific diagnostic markers. Shen et al. have suggested various distinctive molecular alterations in the serum of SARS‐CoV‐2‐infected patients, which resulted in the identification of several specific biomarkers. Such approaches hold promise for predicting the progression from mild to severe conditions.^[^ ^27^ ^]^ Similarly, the use of machine learning has yielded a remarkable 94% accuracy in identifying critical biomarkers related to long COVID‐19.^[^ ^28^ ^]^ Another recent study has also uncovered 22 long COVID‐19 protein markers, providing valuable insights for the early identification of high‐risk individuals.^[^ ^29^ ^]^ In this study, we pioneered the use of metabolomics and lipidomics with machine learning and discovered serum metabolites and lipids as biomarkers that are differential and distinct to PRRSV infection. Of them, we confirmed that LPA serves as a decisive biomarker of PRRSV infection, thus presenting implications for the clinical diagnosis of PRRSV infections and providing guidance to discover biomarkers for other viral infections.

An aspect of the host‐virus interaction is the tendency of viruses to reprogram the metabolic activity of the host cells, providing opportunities to develop novel antiviral drugs.^[^ ^17^, ^22^, ^30^, ^31^ ^]^ In the context of PRRSV infection, studies suggest that PRRSV hijacks tryptophan‐polyamine metabolism to promote viral proliferation,^[^ ^32^ ^]^ which is in agreement with the pathway enrichment data derived from our in vivo infection model. PRRSV activates the AMP activated protein kinase (AMPK)‐ acetyl‐CoA carboxylase 1(ACC1) signaling pathway to regulate fatty acid synthesis in cultured cells, which is unfavorable for PRRSV proliferation.^[^ ^33^ ^]^ However, no significant changes in fatty acid metabolism were observed in our study involving clinical pig cohorts and in vivo infection models. Such discrepancies may occur due to the fact that PRRSV‐infected cells may not accurately reflect the metabolic alterations in the infected pig's body in clinical settings. To the best of our knowledge, our study presented for the first time a comprehensive metabolite and lipid profile changes within PRRSV‐infected pigs. These findings may provide novel insights into PRRSV replication biology and could lead to the development of potentially new antiviral approaches.

LPA is characterized as a small and structurally simple biologically active phospholipid. LPA is involved in various biological processes, including the regulation of wound healing, blood clotting, blood pressure maintenance, cell survival, proliferation, differentiation, migration, morphology, and adhesion.^[^ ^34^, ^35^, ^36^ ^]^ A recent study has indicated that the binding of LPA to one of its receptors LPAR1 induces Rho‐associated coiled‐coil‐forming protkinase 1/2 (ROCK1/2), leading to the inactivation of IRF3 and dampening of IFN responses against viruses like Zika virus and SARS‐CoV‐2.^[^ ^25^ ^]^ However, the role of LPA in PRRSV infection remained unknown so far. In this study, we elucidated that PRRSV reprogrammed host metabolites and lipids by ATX‐mediated upregulation of LPA level which subsequently induces a proviral effect. Targeting LPA effectively inhibited viral replication at both in vitro and in vivo levels. The LPA‐mediated proviral effect was independent of LPAR1, indicating the engagement of other upstream mechanisms. One possible mechanism was found through increased ATX expression; however, the precise mechanism by which PRRSV modulates the ATX‐LPA axis remained unprobed in this study and requires further investigations. Since several genotypes of PRRSV contribute to pig mortality in the natural environment, additional studies are needed to determine whether targeting LPA has a universal effect on all PRRSV genotypes.

PRRSV exerts significant immunosuppressive effects in pigs. During PRRSV infection, the virus undergoes prolonged replication within the host without triggering pronounced innate immune responses, particularly the type 1 IFN response. To date, most studies have predominantly focused on the suppression of the IFN pathway from the perspective of PRRSV infection in general or viral protein specifically. For instance, PRRSV infection can suppress the IFN‐β production by interfering with the mitochondrial antiviral signaling protein (MAVS) activation in the RIG‐I signaling pathway.^[^ ^37^ ^]^ PRRSV non‐structural protein 4 (Nsp4) and Nsp11 proteins selectively target RIG‐I and MAVS to inhibit type I IFN signaling cascade.^[^ ^38^, ^39^ ^]^ However, the regulation of IFN or other innate immune pathways by the host metabolites or lipids is unknown. We unveiled that a phospholipid LPA, increased upon PRRSV infection, negatively regulates the IFN‐β production via suppressing the RIG‐I. Our findings shed light on a novel immunosuppressive mechanism employed by PRRSV, guiding further exploration into the molecular intricacies of PRRSV replication.

In summary, by utilizing a machine learning approach and integrating clinical pig cohorts with in vivo and in vitro infection models, we identified serum biomarkers of metabolic and lipid origins that are capable of distinguishing PRRSV infection. This led to unveil LPA as an inducer of PRRSV replication and associated disease pathogenesis which upon targeting showed a promising therapeutic effect. This work could serve as a pivotal reference for the clinical diagnosis of PRRSV infection and the establishment of novel therapy to treat PRRSV infections.

## 4 Experimental Section

### Detection of Untargeted Metabolome and Lipidome

Metabolomic detection was performed using the Q Exactive HF high‐resolution mass spectrometer at BGI Genomics (Shenzhen, China), employing liquid chromatography‐tandem mass spectrometry (LC‐MS/MS). Data were collected in both positive and negative ion modes to enhance metabolite coverage. Untargeted metabolomic data were processed with Compound Discoverer 3.1, including peak extraction, alignment, and compound identification. Lipidomic data were analyzed using LipidSearch 4.1 for intelligent peak extraction, lipid identification, and peak alignment. Compound identification utilized multiple databases, including the BGI Library (a proprietary standard product library), mzCloud, and ChemSpider (encompassing HMDB, KEGG, LipidMaps analyses).

### Data Preprocessing and Quality Control

Results from Compound Discoverer 3.1 and LipidSearch 4.1 were imported into metaX for preprocessing, which involved: 1) Data normalization using the Probabilistic Quotient Normalization (PQN) method to obtain relative peak areas; 2) Batch effect correction with the QC‐RLSC (Quality Control‐based Robust LOESS Signal Correction); 3) Removal of compounds with a Coefficient of Variation (CV) over 30% in all QC samples. Data quality was evaluated through the reproducibility of quality control (QC) samples, including chromatogram overlap, PCA, peak numbers, and peak response intensity differences.

### Differential Metabolites and Lipids Screening

Differential metabolites and lipids were screened using the first two principal components' VIP values from the PLS‐DA (Partial Least Squares Method‐Discriminant Analysis) model, combined with univariate analysis of fold‐change and Student's t‐test. Criteria for differential metabolites and lipids were: VIP ≥ 1 in the first two PLS‐DA components, Fold‐Change ≥ 1.2 or ≤ 0.83, and q‐value < 0.05.

### Functional Enrichment Analysis of Differential Metabolites and Lipids

Differential metabolites and lipids were subjected to functional identification and pathway enrichment analysis using the KEGG database^[^ ^40^ ^]^ and MetaboAnalyst website (https://www.metaboanalyst.ca).^[^ ^41^ ^]^ The information of software and databases used in functional analysis were listed in Table S5 (Supporting Information).

### Correlation Analysis Between Differential Metabolites/Lipids and Cytokine Levels

The correlation between differential metabolites/lipid molecules and cytokine expression changes was calculated using the Spearman correlation coefficient in the R package corrplot.^[^ ^42^ ^]^ The information of software used for correlation analysis were listed in Table S5 (Supporting Information).

### Cell Culture

MARC‐145 cells were cultured in Dulbecco's modified Eagle's medium (DMEM) supplemented with 10% of fetal bovine serum (FBS) and 1% penicillin‒streptomycin (PS). iPAM cells were cultured in RPMI 1640 medium supplemented with 10% FBS and 1% PS. All cells were incubated at 37 °C with 5% of CO_2_ in an incubator. The information of regents used for cell culture could be found in Table S5 (Supporting Information).

### Propagation and Measurement of PRRSV

MARC‐145 cells were grown in T175 cell culture flasks to full confluency. Before infection, cultured cells were washed thrice with 1× PBS gently. Subsequently, cells were infected with highly pathogenic PRRSV (HP‐PRRSV) Li11 strain,^[^ ^43^ ^]^ diluted in an infection medium (DMEM supplemented with 2% FBS), for a period of 1 h at 37 °C with 5% CO_2_. Post‐viral incubation, the cells were washed with 1× PBS and maintained in a fresh infection medium. Upon observing significant cytopathy, cells were subjected to three freeze‐thaw cycles at −80 °C and centrifuged at high speed (10,000 rpm) at 4 °C for 10 min to collect the supernatant, which was then stored at −80 °C. A 50% tissue culture infectious dose (TCID_50_) of PRRSV was determined by seeding MARC‐145 cells in a 96‐well plate at a density of 1×10^4^ cells/well, incubating them until they become fully confluent, and then treating them with PRRSV (serially diluted in the infection medium) at 37 °C and 5% CO_2_. After 1 h of virus absorption, cells were washed with 1× PBS and continued cultivation with a maintenance medium (DMEM supplemented with 2% FBS) at 37 °C with 5% CO_2_ for 96–144 h. Cell cytopathy for each dilution was recorded daily, and the TCID_50_ of PRRSV was calculated using the Reed‐Muench method. The information of regents used for PRRSV propagation were listed in Table S5 (Supporting Information).

### CCK‐8 Assay for Drug Cytotoxicity

iPAMs were seeded in a 96‐well plate at a density of 1×10^4^ cells/well and cultured at 37 °C with 5% CO_2_ until they reach full confluency. Different concentrations of LPA (5 µM to 500 µM) were prepared in RPMI 1640 medium and added to the PBS‐washed cells, followed by incubation at 37 °C for 48 hours. Later, the cell supernatants were replaced with a CCK‐8 solution, and the cells were incubated for an additional 1 h. Absorbance was measured at 450 nm by using the ELISA microplate reader (BioTek, USA), and cell viability was calculated. This procedure was repeated independently three times for averaging. The cytotoxicity assessment of HA130 and Ki16425 followed a similar procedure, ensuring the DMSO concentration in the cell culture medium was kept below 0.1%. The information of regents used for CCK‐8 were listed in Table S5 (Supporting Information).

### ELISA for LPA Concentration Measurement

LPA concentrations in cell culture supernatants and animal serum samples were determined using the Signalway Antibody (SAB) LPA ELISA kit. LPA standards provided in the kit were dissolved, shaken vigorously, and serially diluted following the manufacturer's instructions. These standards, along with the cell culture supernatants or serum samples, were added to a 96‐well plate. After adding the HRP‐conjugate to each well and incubating at 37 °C for 1 h, the wells were washed, and a substrate solution was added. The reaction was stopped upon color development, and absorbance was measured at 450 nm by using the ELISA microplate reader (BioTeck, USA) to calculate the LPA concentration. The information of regents used for ELISA assays were listed in Table S5 (Supporting Information).

### Western Blot

Cells were lysed on ice for 10 min with a radio immunoprecipitation assay (RIPA) lysis buffer, and the lysates were centrifuged at 12,000 g and 4 °C for 5 min to collect the supernatant. Protein concentration in each sample was measured using the BCA protein assay kit. Protein samples were prepared by mixing the supernatant with Omni‐Easy Protein Sample Loading Buffer and were subjected to boiling at 100 °C for 3 min. SDS‐PAGE electrophoresis was performed, and proteins were transferred onto the PVDF membranes using a Mini Trans‐Blot Cell (BioRad, USA). Membranes were blocked at room temperature for 2 h in a blocking buffer containing 1% bovine serum albumin and 0.05% Tween‐20 in 1× PBS, followed by incubation with indicated primary antibodies overnight at 4 °C. After washing thrice with a 0.05% Tween‐20 solution in 1× PBS, membranes were incubated with indicated secondary antibodies at room temperature for 1 h. Membranes were visualized using the enhanced chemiluminescence (ECL) reagent. The information of antibodies and regents used for western blot were listed in Table S5 (Supporting Information).

### Indirect Immunofluorescence Assay (IFA)

iPAMs were seeded in a 24‐well plate at a density of 5×10^4^ cells/well, and cultured until became full confluency, followed by drug and virus treatment as indicated. Treated cells were fixed with paraformaldehyde for 15 min at room temperature. Then, cells were permeabilized with Triton X‐100 for 15 min at room temperature., Subsequently, cells were washed thrice with 1× PBS and were blocked in 1% BSA solution in 1× PBS for a period of 1 h at room temperature. Thereafter, cells were incubated with primary mouse anti‐PRRSV‐N protein antibody for 2 h at room temperature, washed thrice with 1× PBS, and incubated with FITC‐conjugated goat anti‐mouse secondary antibody for 1 h at room temperature. Nuclei were stained with DAPI for another 10 min at room temperature, and cells were visualized using a confocal fluorescence microscope (Nikon, Eclipse 80i). The information of antibodies and regents used for IFA were listed in Table S5 (Supporting Information).

### Isolation of Porcine Alveolar Macrophages (PAMs) from Bronchoalveolar Lavage Fluid

Animals were subjected to anesthetize with Zoletil and exsanguinate. Lungs were carefully exposed, and a cannula was inserted into the trachea. A sterile PBS was gently infused into the lungs, ensuring even distribution across the lung lobes. The PBS was then slowly aspirated back, collecting the bronchoalveolar lavage fluid which contains a mixture of lung cells and alveolar contents. The collected lavage fluid was then transferred to sterile centrifuge tubes and centrifuged at 300× g for 10 min at 4 °C to pellet the cells. The supernatant was carefully aspirated and discarded by without disturbing the cell pellet. The pellet, primarily consisting of PAMs, was gently resuspended in an RPIM 1640 medium. This suspension of PAMs was further purified by layering over a density gradient medium and centrifuging at 400× g for 20 min at room temperature. The layer containing the PAMs was carefully aspirated and transferred to a new tube, washed with the culture medium, and centrifuged under the same conditions to obtain a purified PAMs pellet. The final PAMs pellet was finally resuspended in a fresh cell culture medium suitable for subsequent experimental procedures. The whole process was performed under aseptic techniques to ensure cell viability and prevent contamination.

### Hematoxylin and Eosin (H&E) Staining of Tissue Sections

Tissue samples were preserved and fixed in a 10% formalin solution to maintain cellular morphology at 4 °C. Then tissues were trimmed, washed under running water for 30 min, and dehydrated in a graded ethanol series (70%, 80%, 90%, 95%, and 100%), each step lasting 40 min, followed by clearing in xylene. The tissues were then embedded in paraffin wax in a warming oven. Once infiltrated with wax, they were embedded, cooled, and solidified into blocks. These blocks were sectioned into thin slices of 5–8 µm using a microtome. The sections, if crinkled, were flattened in warm water and mounted on slides for drying at 45 °C. The sections were subjected to dewaxing in a gradient of xylene and rehydrating through a series of alcohols. Subsequently, sections were stained with hematoxylin, rinsed, differentiated in hydrochloric acid alcohol, blued in ammonia water, and stained with eosin before final washing. After clearing in ethanol and xylene, the slides were sealed with neutral gum for observation. The information of regents used in H&E were listed in Table S5 (Supporting Information).

### TUNEL Staining of Tissue Sections

The process of embedding and dewaxing tissue sections was similar to that described for H&E staining. TUNEL Staining was performed by following the manufacturer's protocol. In brief, the sections were treated with 1% Triton X‐100 for permeabilization for 5 min at room temperature, followed by washing with PBS. They were then treated with Proteinase K from the TUNEL kit, washed, and dried. TdT enzyme reaction solution was applied to each sample, which was then incubated in a humidified, light‐protected box. After washing, Streptavidin‐TRITC was added and incubated in the dark, followed by PBS washes. The nuclei were counterstained with DAPI, washed with PBS thrice and visualized utilizing a slide scanner (ZEISS Axioscan 7, Germany). The information of antibodies and regents used for TUNEL assay were listed in Table S5 (Supporting Information).

### Plasmids and small interference RNAs (siRNAs)

The pig *ENPP2* and *RIG‐I* gene was amplified through reverse transcription PCR from total RNA extracted from iPAM cells using gene‐specific primers and subsequently cloned into a pUC57 vector containing a C‐terminal HA tag. The plasmid was sequenced to confirm that no errors were introduced during PCR amplification. Cultured iPAM cells were transfected with 1 µg of plasmids or 100 pmol of siRNAs (small interference RNAs) using jetPRIME according to the manufacturer's instructions. The information of regents and oligonucleotides used for over‐expression and siRNAs were listed in Table S5 (Supporting Information).

### Detection of Cytokines and Chemokines

Cytokine and chemokine levels were quantified using the Luminex 200 high‐throughput liquid protein chip as described previously,^[^ ^44^ ^]^ following a streamlined protocol. Initially, reagents were prepared, including the dilution of wash buffer to 1× concentration with deionized water, and the preparation of bead and detection antibody mixtures to the 1× concentrations. Standard samples were prepared by brief centrifugation and mixing with universal assay buffer, followed by a four‐fold serial dilution in PCR 8‐strip tubes. Beads were vortexed and allocated to a 96‐well plate, which then underwent magnetic separation for washing. Each well was subsequently filled with a mixture of universal assay buffer and added with experimental or standard samples. The plate was incubated with agitation at room temperature, then stored at 4 °C. The procedure continued with the addition of detection antibodies post‐washing, followed by incubation, and then the addition of streptavidin‐phycoerythrin (SA‐PE). After a final washing step, a reading buffer was added, and the Luminex 200 instrument was used for data acquisition. Data analysis was conducted using a five‐parameter nonlinear regression method for standard curve fitting and concentration calculations. The information of regents used in Luminex‐based cytokines and chemokines detection could be found in Table S5 (Supporting Information).

### Animal Experiment of PRRSV‐Challenged Model

An animal infection model was established using 4‐week‐old commercial piglets, which all tested negative for PRRSV, porcine circovirus type 2 (PCV2), pseudorabies virus (PRV), and classical swine fever virus (CSFV) antibodies and antigens via ELISA and RT‐qPCR methods. The piglets were randomly divided into two groups (*n* = 5 per group) and acclimatized in separate environments with free access to water and food for two days. Piglets in one group were intramuscularly injected with 2×10^6^ TCID_50_ of HP‐PRRSV Li11 virus, while the other group received an equivalent volume of DMEM as the mock control. Continuous monitoring of body temperature and weight was conducted post‐infection. Clinical symptoms in the infected group were recorded and scored as follows: sneezing (normal: 0 points, sneezing: 1 point); coughing (normal: 0 points, coughing: 1 point); spirit (normal: 0 points, lethargy/drowsiness: 1 point); appetite (normal: 0 points, reduced: 1 point, severely reduced/not eating: 2 points); skin (normal: 0 points, cyanosis: 1 point); and breathing (normal: 0 points, mild difficulty: 1 point, obvious abdominal breathing: 2 points, continuous/intermittent mouth breathing: 3 points). Animals were subjected to anesthetize with Zoletil and exsanguinate. Blood samples were collected on days 3, 7, 10, and 21 post‐infection for untargeted metabolomic and lipidomic analysis. The experiment was conducted under supervision of the Institutional Animal Care and Use Committee (IACUC) of Sun Yat‐sen University (IACUC DD‐17‐0403), adhering strictly to the regulations and guidelines set forth by this committee. The information of regents and animals were listed in Table S5 (Supporting Information).

### Animal Experiment of HA130 Treatment Model

About four‐week‐old commercial piglets tested negative for PRRSV, PCV2, PRV, and CSFV antibodies and antigens via ELISA and RT‐qPCR, were randomly divided into four groups (*n* = 12 per group). The groups were: (1) Mock control, each piglet injected with an equivalent volume of DMEM with concurrent injection of solvent carrier at the treatment initiation; (2) Drug control, injected with DMEM, followed by treatment with HA130 solution during the treatment phase; (3) Infection group, intramuscularly injected with virus and with solvent carrier during treatment; and (4) Infection plus treatment group, injected with virus and treated with HA130 solution intramuscularly for three consecutive days upon the onset of fever symptoms. After transportation to the disinfected experimental site, piglets were divided into four groups and acclimatized to their respective environments with free access to food and water for two days. On the third day, the third and fourth groups were infected by intramuscular injection of 2×10^6^ TCID_50_ HP‐PRRSV Li11 virus, while the first and second groups received equivalent volumes of DMEM. Daily monitoring of rectal temperature was conducted post‐infection, and treatment with HA130 was initiated in the second and fourth groups once fever symptoms (≥40 °C) were observed. HA130 solution was administered intramuscularly at a dosage of 1 mg·kg^−1^ body weight for three consecutive days. Continuous monitoring of rectal temperature and clinical symptoms, including weight, skin condition, feeding behavior, and mental state, was carried out. Animals were subjected to anesthetize with Zoletil and exsanguinate. Serum samples were collected on 1, 3, 5, and 7 days post‐drug treatment. Three piglets from each group were randomly selected for necropsy to collect lung, spleen, and inguinal lymph node samples for subsequent experimental analysis. The experiment was conducted under the same strict supervision as the previous experiment, ensuring compliance with all relevant regulations and guidelines. The information of regents and animals were listed in Table S5 (Supporting Information).

### Preparation of Samples for RNA‐Sequencing

Six four‐week‐old commercial piglets, confirmed negative for PRRSV, PCV2, PRV, and CSFV antibodies and antigens via ELISA and RT‐qPCR, were randomly allocated into two groups (*n* = 3 per group). One group was subjected to infection by intramuscular injection of 2×10^6^ TCID_50_ of the HP‐PRRSV Li11 virus, while the other group received an equivalent volume of DMEM as a control. Seven days post‐infection, all piglets were euthanized with Zoletil, and bronchoalveolar lavage fluid was collected for the isolation of porcine alveolar macrophages (PAMs). The isolated PAMs were then processed for RNA sequencing on the DNBSEQ‐T7 platform. The experiment was conducted under the same strict supervision as previous description. The information of regents and animals were listed in Table S5 (Supporting Information).

### Preparation of Samples for Proteomics

iPAM cells were seeded in 150 mm culture dishes at a density of 5×10^6^ cells/dish. The dishes were divided into four groups, each with three biological replicates, and allowed to grow until reaching 90% confluency. One group served as a blank control with no treatment, one group was pre‐treated with HA130 for 1 h before the growth medium was replaced, one group was infected with PRRSV at an MOI of 0.1, and the final group was pre‐treated with HA130 for 1 h before being infected with PRRSV at an MOI of 0.1. At 24 h post‐treatment, cells from each group were harvested for proteomic analysis using a mass spectrometry approach.

### Data Analysis and Visualization

Data analysis and visualization, including differential metabolites and lipids screening, volcano plots and correlation analysis were performed in R language^[^ ^45^ ^]^ within R Studio.^[^ ^46^ ^]^ The heatmaps were generated with TBtools.^[^ ^47^ ^]^ The bar plots were generated in GraphPad Prime. Finally, the integrated figures were plotted together using Adobe Illustrator. The information of software used for data analysis and visualization could be found in Table S5 (Supporting Information).

### Statistical Analysis

All data in this study were presented as the mean ± SEM. Statistical analyses were conducted using GraphPad Prism (Version 8.4.3). Error bars indicate the SEM. The Mann‐Whitney test was employed to assess the statistical significance of differences between two groups. For testing the significance of predicting results in machine learning models, Chi‐square test was used to calculate the p‐values. For correlation analysis, Spearman's correlation coefficient was calculated using Spearman's correlation test, and the p‐value was calculated using Pearson's Chi‐square test. Significance levels were denoted as follows: * for 0.01 < *p*‐value < 0.05, ** for 0.001 < *p*‐value < 0.01, and *** for 0.0001 < *p*‐value < 0.001.

## Conflict of Interest

The authors declare no conflict of interest.

## Author Contributions

H.Z., F.H., and O.P. contributed equally to this work. H.Z. and Y.C. designed and supervised the study. F.H., Y.H., and G.H. performed the experiments. H.Z., O.P., F.H., Y.H., and C.Z. analyzed and visualized the data. H.Z., O.P., and U.A. wrote and revised the manuscript. Q.X., B.Z., W.L., C.L., and C.X. provided the ideas and suggestions. M.C. and X.W. provided the technical supports. Y.W. and Q.L. helped to collect clinical samples. All authors have given approval to the final version of the manuscript.

## Supporting information

Supporting Information

Supplementary Table S1

Supplementary Table S2

Supplementary Table S3

Supplementary Table S4

Supplementary Table S5

## Data Availability

The data that support the findings of this study are available from the corresponding author upon reasonable request.

## References

[1] J. Zhu , X. He , D. Bernard , J. Shen , Y. Su , A. Wolek , B. Issacs , N. Mishra , X. Tian , A. Garmendia , Y. Tang , J. Virol. 2023, 97, e0005423. 37133376 10.1128/jvi.00054-23 PMC10231194

[2] X. Yu , Q. Sun , X. Ku , D. He , Z. Li , A. H. Ghonaim , S. Fan , Q. He , Vet. Med. Sci. 2021, 7, 175. 32583623 10.1002/vms3.289 PMC7840206

[3] G. Saade , C. Deblanc , J. Bougon , C. Marois‐Crehan , C. Fablet , G. Auray , C. Belloc , M. Leblanc‐Maridor , C. A. Gagnon , J. Zhu , M. Gottschalk , A. Summerfield , G. Simon , N. Bertho , F. Meurens , Vet. Res. 2020, 51, 80. 32546263 10.1186/s13567-020-00807-8 PMC7296899

[4] C. Levesque , C. Provost , J. Labrie , R. Y. Hernandez , N. J. Burciaga , C. A. Gagnon , M. Jacques , PLoS One 2014, 9, e98434. 24878741 10.1371/journal.pone.0098434 PMC4039538

[5] C. Egli , B. Thur , L. Liu , M. A. Hofmann , J. Virol. Methods 2001, 98, 63. 11543885 10.1016/s0166-0934(01)00358-5

[6] K. J. Sorensen , B. Strandbygaard , A. Botner , E. S. Madsen , J. Nielsen , P. Have , Vet. Microbiol. 1998, 60, 169. 9646448 10.1016/s0378-1135(98)00159-x

[7] K. J. Sorensen , A. Botner , E. S. Madsen , B. Strandbygaard , J. Nielsen , Vet. Microbiol. 1997, 56, 1. 9228677 10.1016/s0378-1135(96)01345-4

[8] M. L. Rotolo , L. Gimenez‐Lirola , J. Ji , R. Magtoto , Y. A. Henao‐Diaz , C. Wang , D. H. Baum , K. M. Harmon , R. G. Main , J. J. Zimmerman , Vet. Microbiol. 2018, 214, 13. 29408024 10.1016/j.vetmic.2017.11.011

[9] C. Fablet , P. Renson , F. Pol , V. Dorenlor , S. Mahe , F. Eono , E. Eveno , M. Le Dimna , D. Liegard‐Vanhecke , S. Eudier , N. Rose , O. Bourry , Vet. Microbiol. 2017, 204, 25. 28532802 10.1016/j.vetmic.2017.04.001

[10] T. Sattler , E. Wodak , S. Revilla‐Fernandez , F. Schmoll , BMC Vet. Res. 2014, 10, 300. 25518885 10.1186/s12917-014-0300-x PMC4276257

[11] A. Kittawornrat , J. Prickett , C. Wang , C. Olsen , C. Irwin , Y. Panyasing , A. Ballagi , A. Rice , R. Main , J. Johnson , C. Rademacher , M. Hoogland , R. Rowland , J. Zimmerman , J. Vet. Diagn. Invest. 2012, 24, 262. 22379043 10.1177/1040638711435679

[12] E. Croft , T. Blackwell , J. Zimmerman , Can. Vet. J. 2020, 61, 420. 32255830 PMC7074113

[13] A. Kittawornrat , Y. Panyasing , C. Goodell , C. Wang , P. Gauger , K. Harmon , R. Rauh , L. Desfresne , I. Levis , J. Zimmerman , Vet. Microbiol. 2014, 168, 331. 24393634 10.1016/j.vetmic.2013.11.035

[14] J. Hu , C. Zhang , Transbound Emerg. Dis. 2014, 61, 109. 23343057 10.1111/tbed.12016

[15] T. Du , Y. Nan , S. Xiao , Q. Zhao , E. M. Zhou , Trends Microbiol. 2017, 25, 968. 28652073 10.1016/j.tim.2017.06.001

[16] F. Long , M. Zhang , X. Yang , X. Liang , L. Su , T. An , G. Zhang , Z. Zeng , Y. Liu , W. Chen , J. Chen , J. Virol. 2022, 96, e0148721. 34787456 10.1128/JVI.01487-21 PMC8826906

[17] Y. B. Baek , H. J. Kwon , M. Sharif , J. Lim , I. C. Lee , Y. B. Ryu , J. I. Lee , J. S. Kim , Y. S. Lee , D. H. Kim , S. I. Park , D. K. Kim , J. S. Kim , H. E. Choy , S. Lee , H. S. Choi , T. F. Osborne , T. I. Jeon , K. O. Cho , Signal Transduct. Target Ther. 2022, 7, 367. 36253361 10.1038/s41392-022-01223-4 PMC9575645

[18] A. Ayari , M. Rosa‐Calatrava , S. Lancel , J. Barthelemy , A. Pizzorno , A. Mayeuf‐Louchart , Baron M. , D. Hot , L. Deruyter , D. Soulard , T. Julien , C. Faveeuw , O. Molendi‐Coste , D. Dombrowicz , L. Sedano , V. Sencio , R. Le Goffic , F. Trottein , I. Wolowczuk , Commun. Biol. 2020, 3, 237. 32409640 10.1038/s42003-020-0965-6 PMC7224208

[19] H. C. Leier , J. B. Weinstein , J. E. Kyle , J. Y. Lee , L. M. Bramer , K. G. Stratton , D. Kempthorne , A. R. Navratil , E. G. Tafesse , T. Hornemann , W. B. Messer , E. A. Dennis , T. O. Metz , E. Barklis , F. G. Tafesse , Nat. Commun. 2020, 11, 3652. 32694525 10.1038/s41467-020-17433-9 PMC7374707

[20] D. Sumbria , E. Berber , M. Mathayan , B. T. Rouse , Front. Immunol. 2020, 11, 594963. 33613518 10.3389/fimmu.2020.594963 PMC7887310

[21] L. Zhou , R. He , P. Fang , M. Li , H. Yu , Q. Wang , Y. Yu , F. Wang , Y. Zhang , A. Chen , N. Peng , Y. Lin , R. Zhang , M. Trilling , R. Broering , M. Lu , Y. Zhu , S. Liu , Nat. Commun. 2021, 12, 98. 33397935 10.1038/s41467-020-20316-8 PMC7782485

[22] N. Xiao , M. Nie , H. Pang , B. Wang , J. Hu , X. Meng , K. Li , X. Ran , Q. Long , H. Deng , N. Chen , S. Li , N. Tang , A. Huang , Z. Hu , Nat. Commun. 2021, 12, 1618. 33712622 10.1038/s41467-021-21907-9 PMC7955129

[23] Y. Pang , C. Li , Y. Wang , J. Liu , G. Su , C. Duan , L. Fang , Y. Zhou , S. Xiao , Vet. Microbiol. 2023, 279, 109674. 36739813 10.1016/j.vetmic.2023.109674

[24] C. M. Malgarin , D. J. Macphee , J. Harding , Front. Mol. Biosci. 2020, 7, 559688. 33363202 10.3389/fmolb.2020.559688 PMC7759636

[25] C. Zhang , W. Li , X. Lei , Z. Xie , L. Qi , H. Wang , X. Xiao , J. Xiao , Y. Zheng , C. Dong , X. Zheng , S. Chen , J. Chen , B. Sun , J. Qin , Q. Zhai , J. Li , B. Wei , J. Wang , H. Wang , Sci. Adv. 2021, 7, eabb5933. 34533996 10.1126/sciadv.abb5933 PMC8448453

[26] T. Matsumiya , D. M. Stafforini , Crit. Rev. Immunol. 2010, 30, 489. 21175414 10.1615/critrevimmunol.v30.i6.10 PMC3099591

[27] B. Shen , X. Yi , Y. Sun , X. Bi , J. Du , C. Zhang , S. Quan , F. Zhang , R. Sun , L. Qian , W. Ge , W. Liu , S. Liang , H. Chen , Y. Zhang , J. Li , J. Xu , Z. He , B. Chen , J. Wang , H. Yan , Y. Zheng , D. Wang , J. Zhu , Z. Kong , Z. Kang , X. Liang , X. Ding , G. Ruan , N. Xiang , et al., Cell 2020, 182, 59. 32492406 10.1016/j.cell.2020.05.032 PMC7254001

[28] J. Klein , J. Wood , J. R. Jaycox , R. M. Dhodapkar , P. Lu , J. R. Gehlhausen , A. Tabachnikova , K. Greene , L. Tabacof , A. A. Malik , M. V. Silva , J. Silva , K. Kamath , M. Zhang , A. Dhal , I. M. Ott , G. Valle , M. Pena‐Hernandez , T. Mao , B. Bhattacharjee , T. Takahashi , C. Lucas , E. Song , D. Mccarthy , E. Breyman , J. Tosto‐Mancuso , Y. Dai , E. Perotti , K. Akduman , T. J. Tzeng , et al., Nature 2023, 623, 139. 37748514 10.1038/s41586-023-06651-y PMC10620090

[29] X. Gu , S. Wang , W. Zhang , C. Li , L. Guo , Z. Wang , H. Li , H. Zhang , Y. Zhou , W. Liang , H. Li , Y. Liu , Y. Wang , L. Huang , T. Dong , D. Zhang , C. Wong , B. Cao , EBioMedicine 2023, 98, 104851. 37924708 10.1016/j.ebiom.2023.104851 PMC10660018

[30] C. S. Palmer , Nat Metab 2022, 4, 1245. 36266542 10.1038/s42255-022-00652-3

[31] S. K. Thaker , J. Ch'Ng , H. R. Christofk , BMC Biol. 2019, 17, 59. 31319842 10.1186/s12915-019-0678-9 PMC6637495

[32] Y. Zhou , Z. Hou , L. Fang , Q. Ke , Y. Xiong , P. Fang , S. Xiao , Vet Microbiol 2020, 250, 108839. 33002680 10.1016/j.vetmic.2020.108839 PMC7501835

[33] S. Long , Y. Zhou , D. Bai , W. Hao , B. Zheng , S. Xiao , L. Fang , Viruses 2019, 11, 1145. 31835577 10.3390/v11121145 PMC6950460

[34] L. A. Broadfield , A. A. Pane , A. Talebi , J. V. Swinnen , S. M. Fendt , Dev. Cell 2021, 56, 1363. 33945792 10.1016/j.devcel.2021.04.013

[35] H. Yoon , J. L. Shaw , M. C. Haigis , A. Greka , Mol. Cell 2021, 81, 3708. 34547235 10.1016/j.molcel.2021.08.027 PMC8620413

[36] L. Geraldo , T. Spohr , R. Amaral , A. Fonseca , C. Garcia , F. A. Mendes , C. Freitas , M. F. Dossantos , F. Lima , Signal. Transduct. Target Ther. 2021, 6, 45. 33526777 10.1038/s41392-020-00367-5 PMC7851145

[37] R. Luo , S. Xiao , Y. Jiang , H. Jin , D. Wang , M. Liu , H. Chen , L. Fang , Mol. Immunol. 2008, 45, 2839. 18336912 10.1016/j.molimm.2008.01.028 PMC7112510

[38] Y. Sun , H. Ke , M. Han , N. Chen , W. Fang , D. Yoo , PLoS One 2016, 11, e0168314. 27997564 10.1371/journal.pone.0168314 PMC5172586

[39] C. Huang , Y. Du , Z. Yu , Q. Zhang , Y. Liu , J. Tang , J. Shi , W. H. Feng , Sci. Rep. 2016, 6, 28497. 27329948 10.1038/srep28497 PMC4916416

[40] M. Kanehisa , S. Goto , Nucleic Acids Res. 2000, 28, 27. 10592173 10.1093/nar/28.1.27 PMC102409

[41] Z. Pang , J. Chong , G. Zhou , M. D. de Lima , L. Chang , M. Barrette , C. Gauthier , P. E. Jacques , S. Li , J. Xia , Nucleic Acids Res. 2021, 49, W388. 34019663 10.1093/nar/gkab382 PMC8265181

[42] T. Wei , V. Simko , R package “corrplot”: Visualization of a Correlation Matrix (Version 0.92), 2022, https://Github.Com/Taiyun/Corrplot.

[43] X. Liu , C. Guo , Y. Huang , X. Zhang , Y. Chen , Infect. Genet. Evol. 2015, 34, 7. 26102162 10.1016/j.meegid.2015.06.021

[44] S. Khalifian , G. Raimondi , G. Brandacher , J. Invest. Dermatol. 2015, 135, 1. 10.1038/jid.2015.36 25785953

[45] T. Rcore , R: A Language and Environment for Statistical Computing, 2023, https://www.R‐project.org.

[46] T. Rstudio , RStudio: Integrated Development for R, 2020, http://www.rstudio.com/2023.

[47] C. Chen , H. Chen , Y. Zhang , H. R. Thomas , M. H. Frank , Y. He , R. Xia , Mol. Plant 2020, 13, 1194. 32585190 10.1016/j.molp.2020.06.009

[48] P. Bardou , J. Mariette , F. Escudie , C. Djemiel , C. Klopp , BMC Bioinformaticss 2014, 15, 293. 10.1186/1471-2105-15-293 PMC4261873 25176396

[49] W. Liebermeister , E. Noor , A. Flamholz , D. Davidi , J. Bernhardt , R. Milo , Proc. Natl. Acad. Sci. USA 2014, 111, 8488. 24889604 10.1073/pnas.1314810111 PMC4060655

[50] A. Subramanian , P. Tamayo , V. K. Mootha , S. Mukherjee , B. L. Ebert , M. A. Gillette , A. Paulovich , S. L. Pomeroy , T. R. Golub , E. S. Lander , J. P. Mesirov , Proc. Natl. Acad. Sci. USA 2005, 102, 15545. 16199517 10.1073/pnas.0506580102 PMC1239896

