# Supplementary material for: Multi‐Omics Analysis by Machine Learning Identified Lysophosphatidic Acid as a Biomarker and Therapeutic Target for Porcine Reproductive and Respiratory Syndrome
Source: Adv Sci (Weinh). 2024 Jul 8;11(34):2402025. doi: 10.1002/advs.202402025 (PMC11425916; doi:10.1002/advs.202402025)
Supplement: Supplementary file 1 — Supporting Information [file ADVS-11-2402025-s001.pdf]

ADVANCED SCIENCE
Open Access

Supporting Information

for *Adv. Sci.*, DOI 10.1002/advs.202402025

Multi-Omics Analysis by Machine Learning Identified Lysophosphatidic Acid as a Biomarker and Therapeutic Target for Porcine Reproductive and Respiratory Syndrome

*Hao Zhang, Fangyu Hu, Ouyang Peng, Yihui Huang, Guangli Hu, Usama Ashraf, Meifeng Cen, Xiaojuan Wang, Qiuping Xu, Chuangchao Zou, Yu Wu, Bibo Zhu, Wentao Li, Qunhui Li, Chujun Li, Chunyi Xue and Yongchang Cao**

Tables list

Table S1. Functional enrichment analysis and its classification of differentially expressed metabolites and lipids in sera originated from clinical samples, related to Figures 1, S1 and 2.

Table S2. Differentially expressed metabolites and lipids in sera originated from clinical samples and animal virus-challenged experiments, related to Figure 2.

Table S3. RNA sequencing and functional analysis of genes differentially expressed in PAMs of piglet origin, related to Figures 3 and S4.

Table S4. Proteomics and functional analysis of proteins differentially expressed in iPAMs, related to Figures 6 and S8.

Table S5. The information of regents, antibodies, oligonucleotides, commercial assays, and software used in this study.

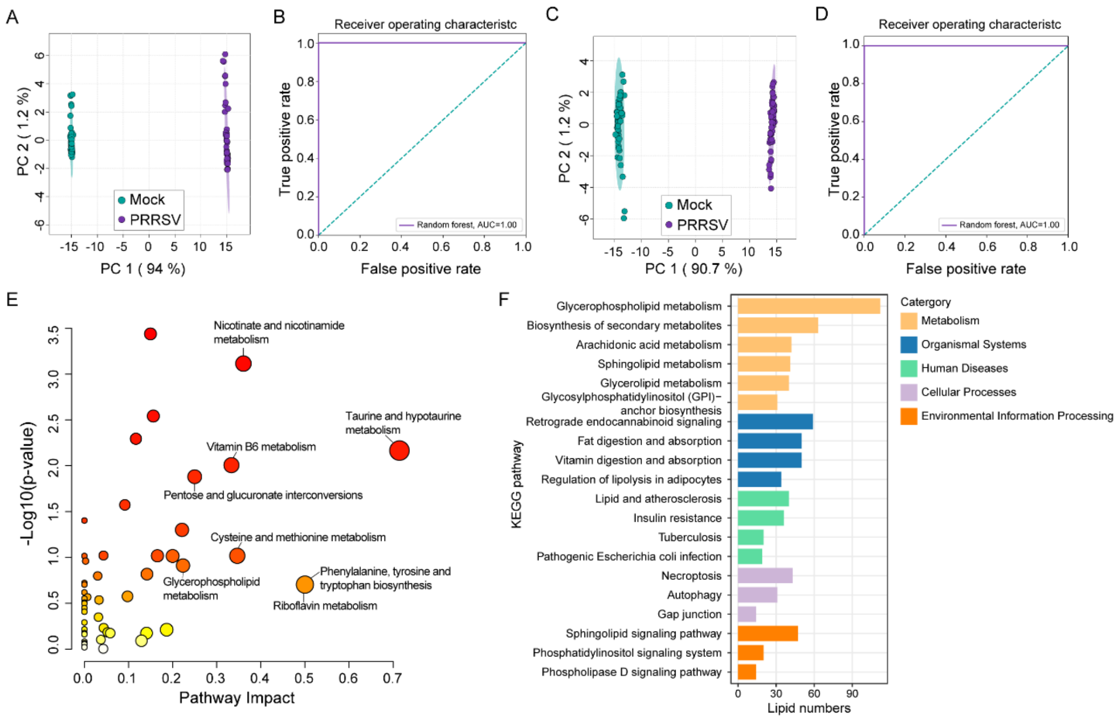

Figure S1. Metabolic and lipidomic data quality control and functional characteristic analysis. A,C) Principal component analysis (PCA) of untargeted metabolomic (A) and lipidomic (C) data. B,D) The receiver operating characteristic (ROC) curve demonstrates machine learning efficiency in analyzing untargeted metabolomic (B) and lipidomic (D) data. E,F) KEGG pathway enrichment of deferentially expressed metabolites and lipids from untargeted metabolomic (E) and lipidomic (F) analyses, respectively. In E, the area of circle denotes enrichment, whereas the color intensity represents statistical significance.

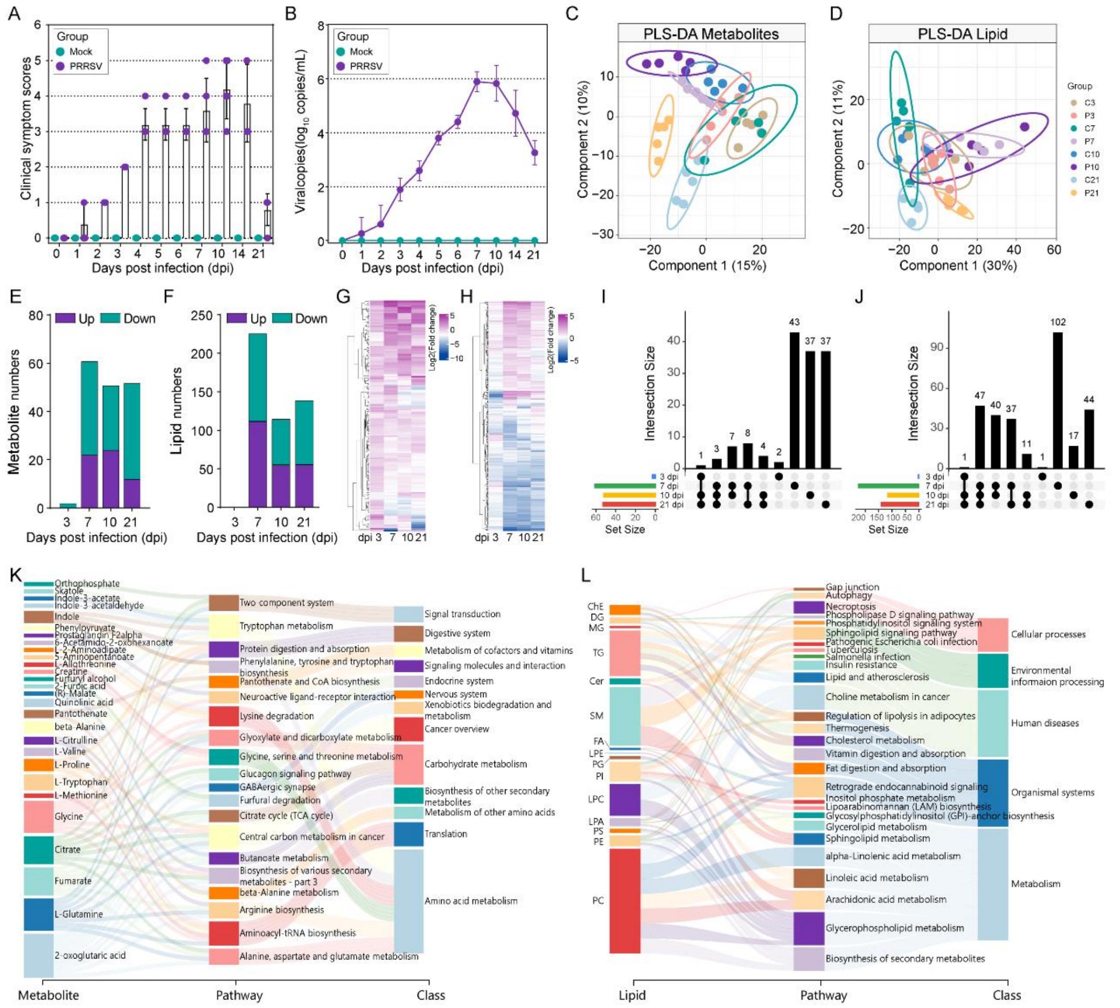

Figure S2. Assessment and metabolomics/lipidomics data analysis in animal virus-challenged experiment. A) Clinical symptom scores of piglets recorded at 0, 1, 2, 3, 4, 5, 6, 7, 10, and 21 days post-infection (dpi). B) Viral load detection in nasal swabs using RT-qPCR. C,D) Principal component analysis (PCA) of untargeted metabolomic (C) and lipidomic (D) data. E,F) Statistical analysis of differentially expressed metabolites (E) and lipids (F) at 3, 7, 10, and 21 dpi. G,H) Heatmaps displaying differentially expressed metabolites (G) and lipids (H) at 3, 7, 10, and 21 dpi. I,J) UpSet diagrams of differentially expressed metabolites (I) and lipids (J) at 3, 7, 10, and 21 dpi, generated with R package UpSetR.[51] K,L) Identification of KEGG pathways associated with differentially expressed metabolites (K) and lipids (L). Diagrams were generated using Charticulator.[52] Data are represented as mean ± SEM.

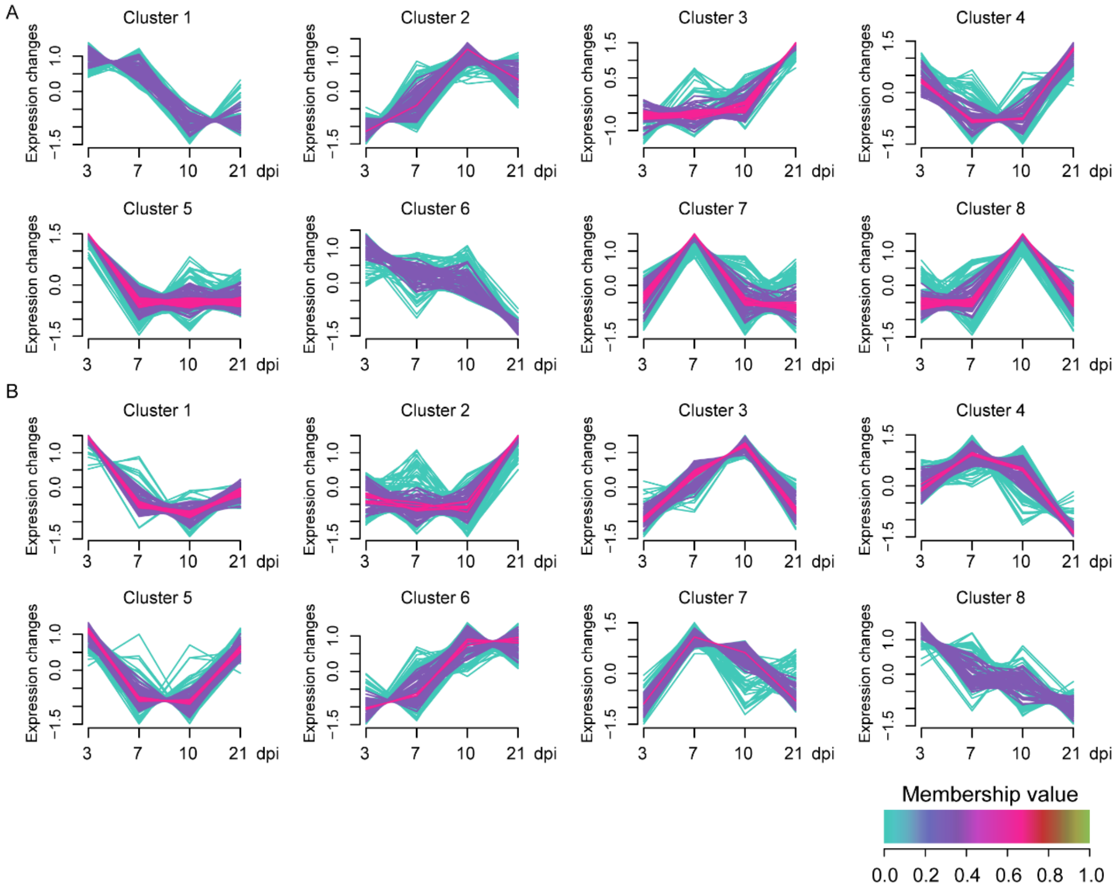

Figure S3. Temporal analysis of metabolites and lipids. Time series analysis of all detected metabolites (A) and lipids (B) at 3, 7, 10, and 21 dpi by using the R package Mfuzz.[53] Both metabolites and lipids were classified into eight clusters by considering their expression tendencies along with the time.

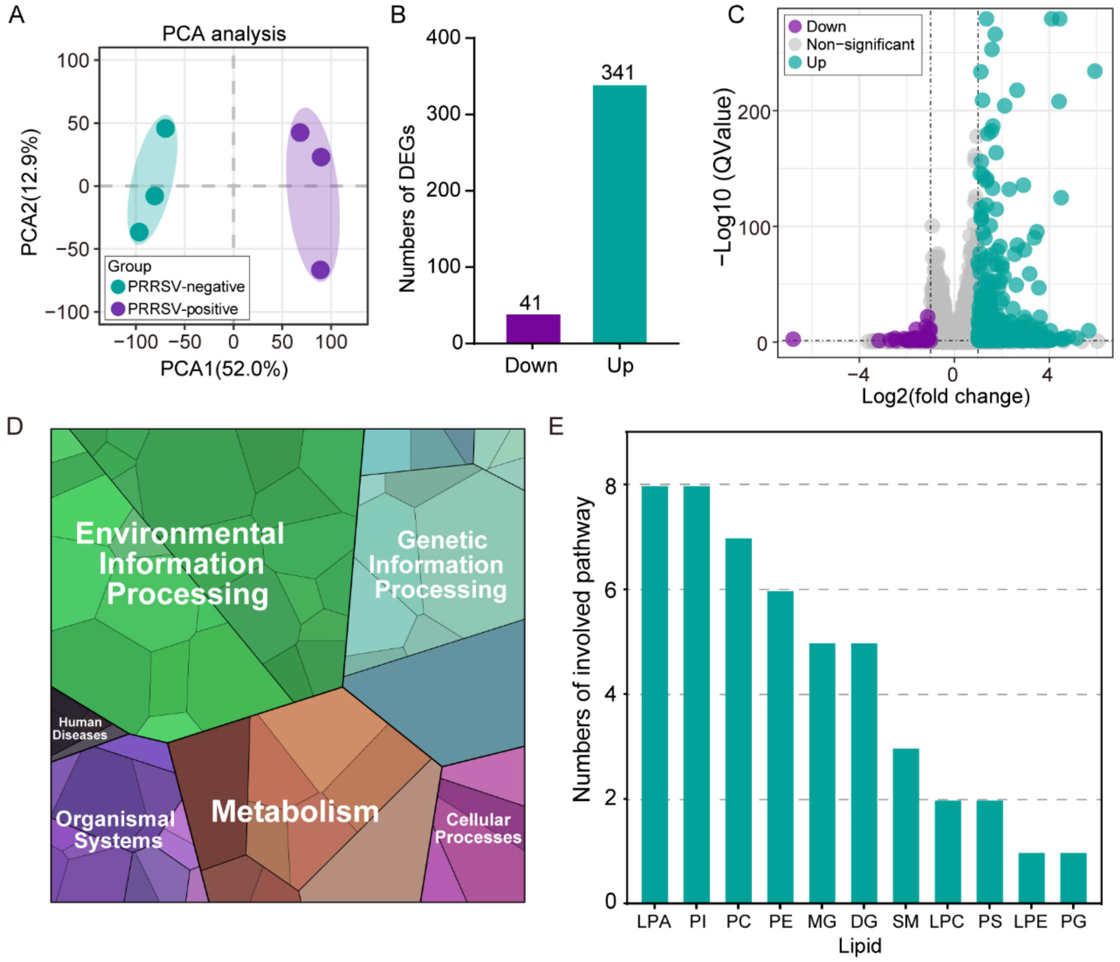

Figure S4. RNA-sequencing analysis of data obtained from an animal PRRSV-challenged experiment. A) Principal component analysis (PCA) of RNA-sequencing data. B,C) Discovery of differentially expressed genes (DEGs). D) KEGG pathway enrichment analysis for all identified DEGs. E) Statistical analysis of pathways influenced by lipids in the animal virus-challenged experiment.

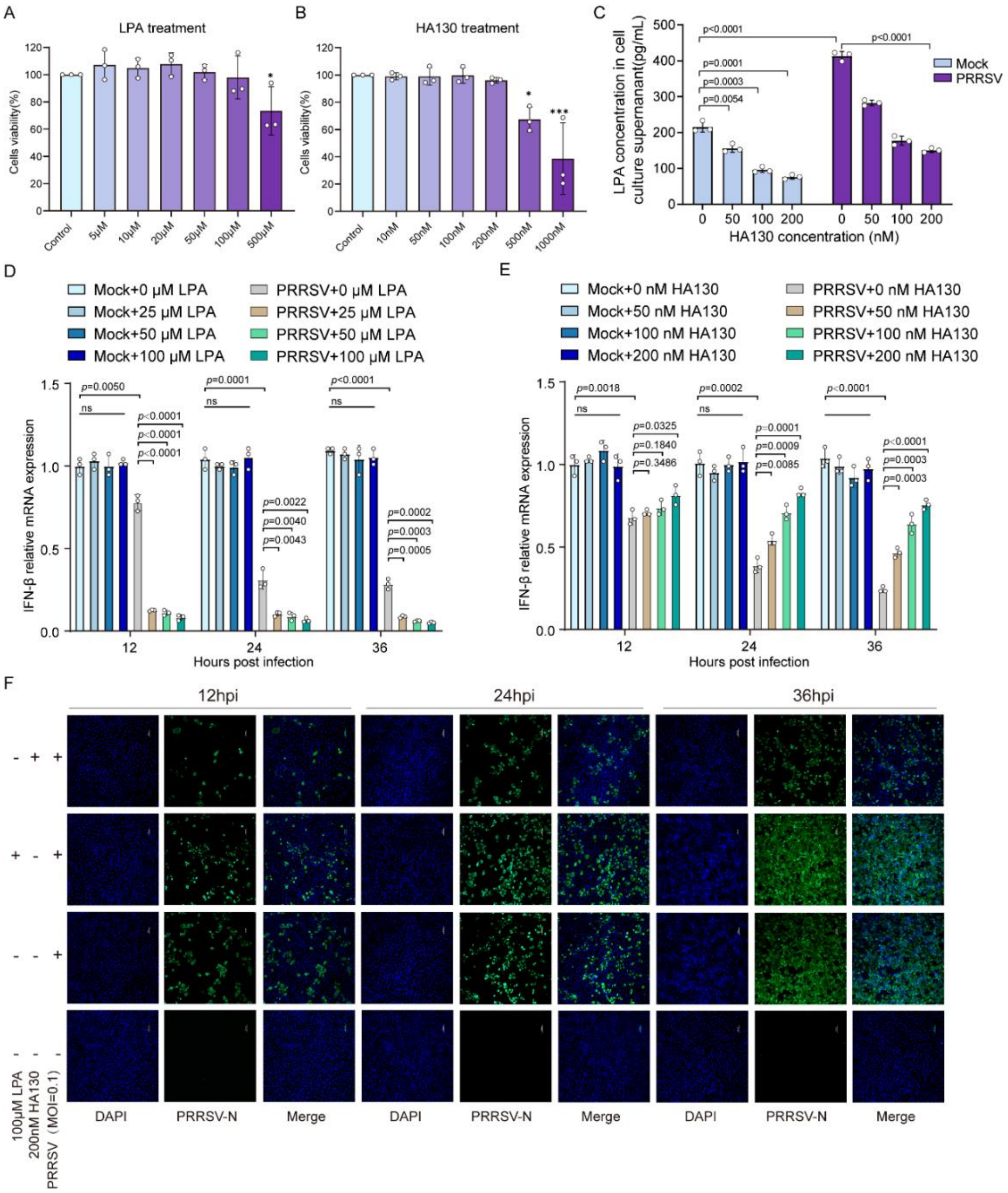

Figure S5. Regulation of PRRSV proliferation by exogenous LPA and LPA inhibitor HA130 *in vitro*. A,B) Assessment of iPAM cytotoxicity by LPA (A) and HA130 (B) using the CCK-8 assay. C) Detection of LPA concentration in iPAMs cell supernatant after HA130 treatment, determined by ELISA. D,E) Dose- and time-dependent quantification of IFN-β mRNA expression in iPAM cells treated with exogenous LPA (D) or HA130 (E) during PRRSV infection, accessed by RT-qPCR. F) Effect of LPA and HA130 on PRRSV-N protein expression, determined by immunofluorescence. The biological experiments were conducted in triplicate. Data are represented as mean ± SEM. Significant comparisons between two groups are made using Mann-Whitney test, significant comparisons among three or more groups are made by one-way ANOVA, with significance levels indicated as ns (not significant), * ($p < 0.05$), ** ($p < 0.01$), and *** ($p < 0.001$).

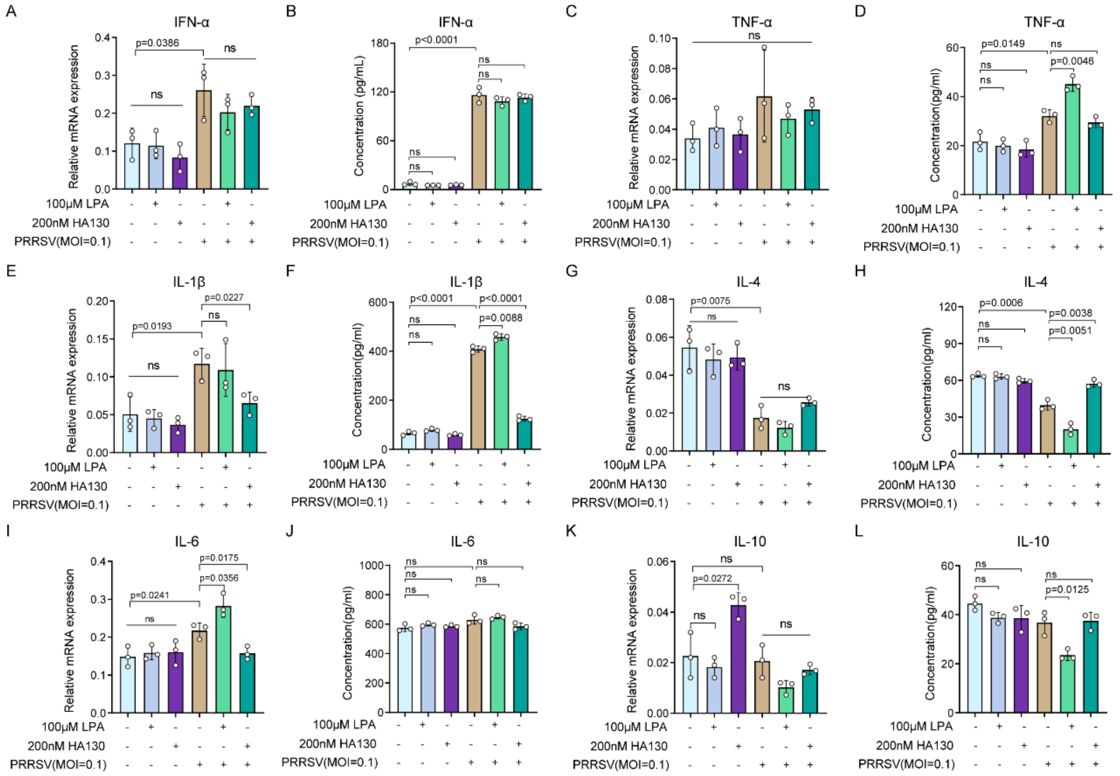

Figure S6. Impact of LPA or HA130 treatment on various cytokine expression levels *in vitro*. A–L) Cultured iPAM cells, treated with indicated concentration of LPA or HA130 for a period of 1 h, were infected with PRRSV at indicated MOI for a period of 24 h. The mRNA and protein expression levels of IFN-α (A and B), TNF-α (C and D), IL-1β (E and F), IL-4 (G and H), IL-6 (I and J), and IL-10 (K and L) were analyzed by RT-qPCR and ELISA, respectively. The biological experiments were conducted in triplicate. Data are represented as mean ± SEM. Significant comparisons between two groups are made using Mann-Whitney test, significant comparisons among three or more groups are made by one-way ANOVA, with significance levels indicated as ns (not significant).

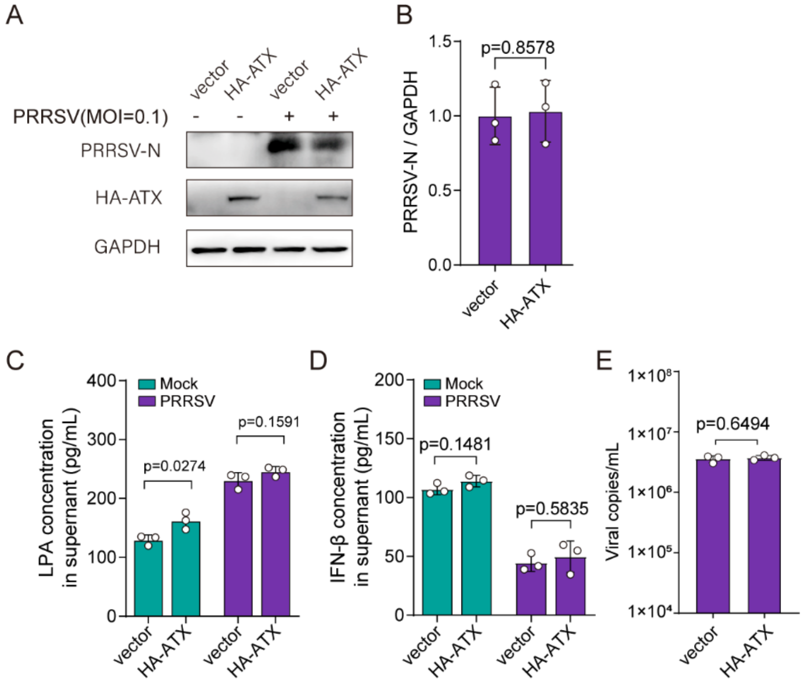

Figure S7. Impact of ATX overexpression on PRRSV replication, LPA Secretion, and IFN-β expression. A–E) Cultured iPAM cells were transfected with 1 μg amount of HA-ATX plasmids or empty vector. At 24 h post-transfection, cells were either uninfected or infected with PRRSV at indicated MOI for a period of 24 h, followed by assessment of PRRSV replication by Western blot (A and B) and RT-qPCR (E), LPA secretion by ELISA (C), and IFN-β secretion by ELISA (D). The biological experiments were conducted in triplicate. Data are represented as mean ± SEM. Significant comparisons between two groups are made using Mann-Whitney test.

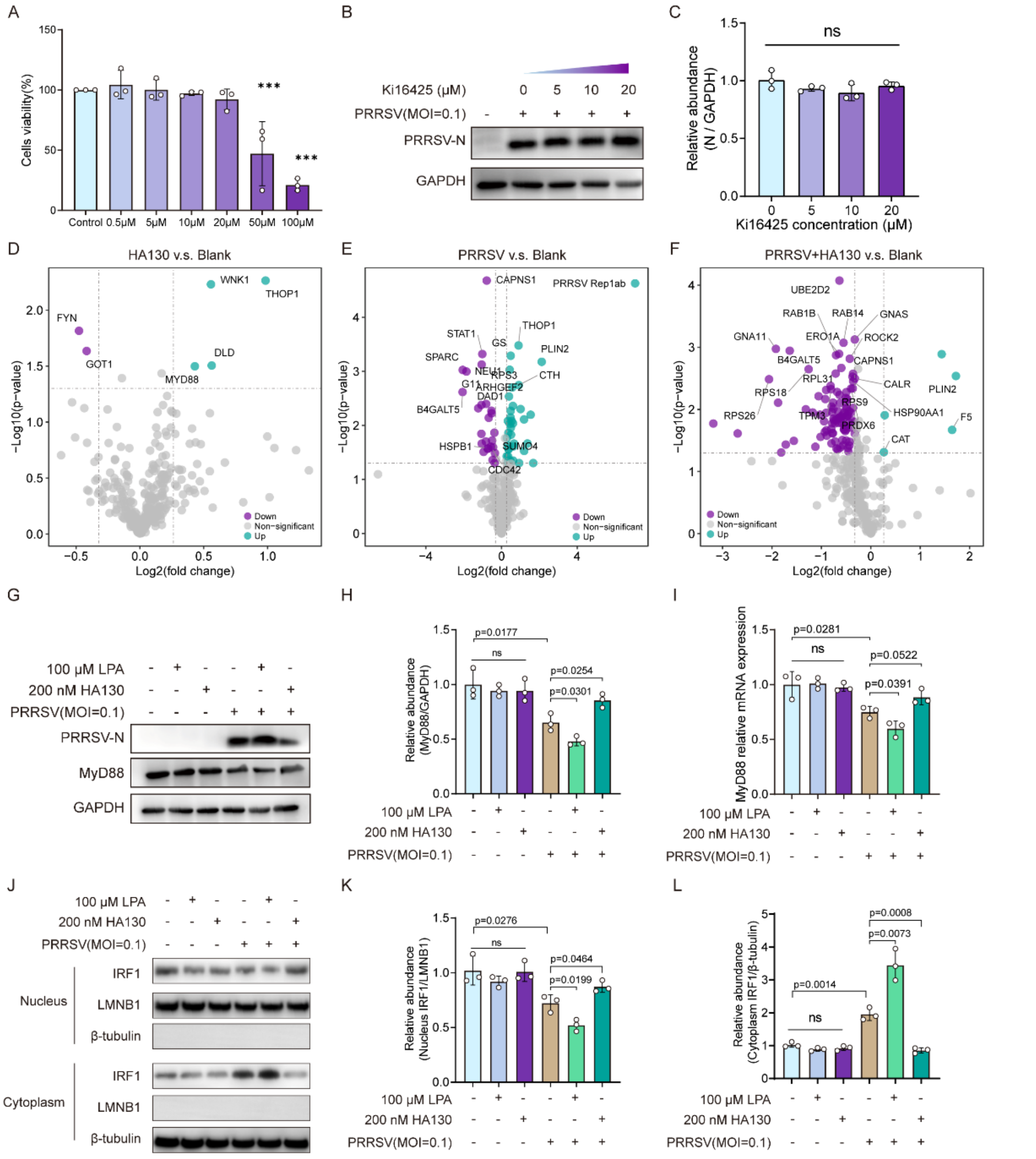

Figure S8. Impact of LPAR1 on PRRSV replication and PRRSV and LPA on MyD88 regulation. A) iPAM cell viability assay with the LPAR1 inhibitor Ki16425. B,C) Ki16425 does not affect PRRSV replication in iPAM cells. D–F) Volcano plots illustrating the differentially expressed proteins (DEPs) detected by proteomics analysis of iPAM cells. G–I) Modulation of MyD88 expression level by PRRSV infection or LPA concentration in iPAM cells. J–L) Regulation of IRF1's nuclear translocation by PRRSV infection or LPA concentration in iPAM cells. In all cell culture experiments, cells were treated with indicated concentrations of Ki16425, LPA, or HA130 for a period of 1 h, followed by PRRSV infection at indicated MOI. At 24 h post-infection, samples were harvested and the protein and mRNA levels of RIG-I and viral protein were determined by Western blot and RT-qPCR. The biological experiments were conducted in triplicate. Data are represented as mean ± SEM. Significant comparisons between two groups are made using Mann-Whitney test, significant comparisons among three or more groups are made by one-way ANOVA, with significance levels indicated as ns (not significant), * ($p < 0.05$), ** ($p < 0.01$), and *** ($p < 0.001$).

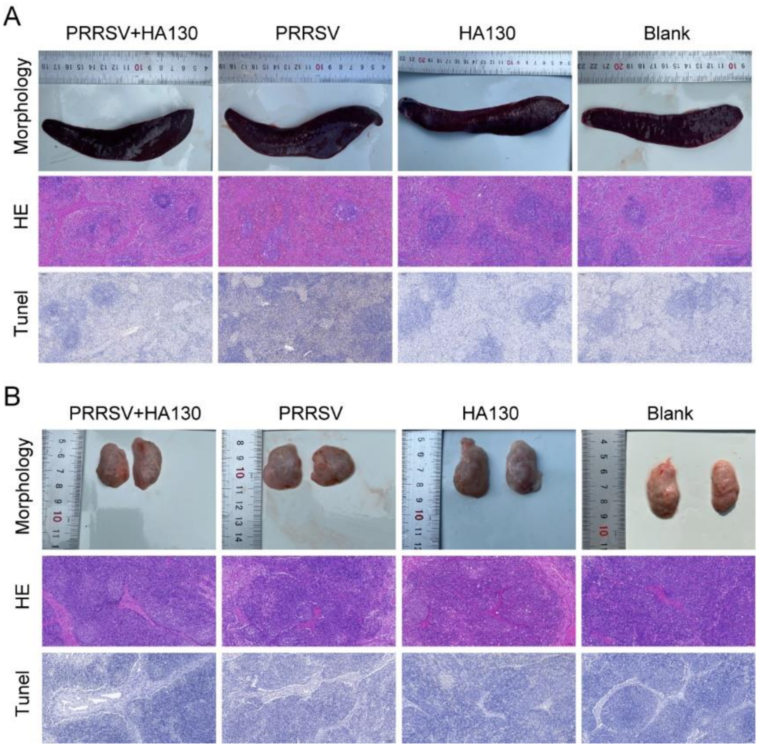

Figure S9. Effect of HA130 treatment in reducing pathological damage and apoptosis in spleens and inguinal lymph nodes of PRRSV-infected piglets. A,B) Morphology, histology, and apoptosis analysis of spleens (A) and inguinal lymph nodes (B) in PRRSV-infected piglets (n = 5 per group). Histological and apoptotic features were visualized by H&E staining and TUNEL assay, respectively.

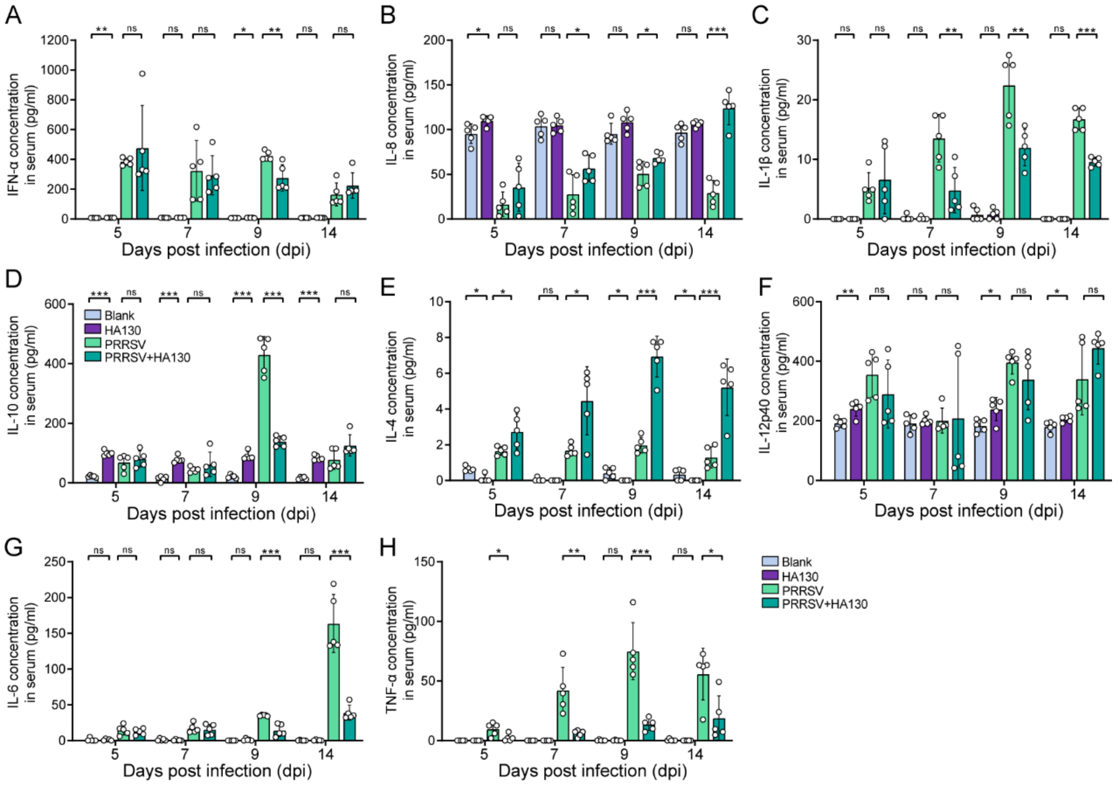

Figure S10. Effect of HA130 treatment on cytokines levels in the serum of PRRSV-infected piglets. The serum levels of IFN-α (A), IL-8 (B), IL-1β (C), IL-10 (D), IL-4 (E), IL-12p40 (F), IL-6 (G), and TNF-α (H) were determined by ELISA. *n* = 5 per group. Data are represented as mean ± SEM. Significant comparisons between two groups are made using Mann-Whitney test, significant comparisons among three or more groups are made by one-way ANOVA, with significance levels indicated as ns (not significant), * ($p < 0.05$), ** ($p < 0.01$), and *** ($p < 0.001$).

[51] J. R. Conway, A. Lex, N. Gehlenborg, Bioinformatics 2017, 33, 2938.
[52] D. Ren, B. Lee, M. Brehmer, IEEE Trans. Vis. Comput. Graph. 2018.
[53] M. E. Futschik, B. Carlisle, J. Bioinform. Comput. Biol. 2005, 3, 965.
Adv.
